# Models of Holomorphic Functions on the Symmetrized Skew Bidisc

**DOI:** 10.1007/s00020-026-02835-z

**Published:** 2026-04-18

**Authors:** Connor Evans, Zinaida A. Lykova, N. J. Young

**Affiliations:** School of Mathematics, Statistics and Physics, Newcastle University, Newcastle upon Tyne, NE1 7RU U.K.

**Keywords:** Schur class, Symmetrized bidisc, Hilbert space models, 32A10, 30E05, 47B99, 47N70

## Abstract

The purpose of this paper is to develop the theory of holomorphic functions with modulus bounded by 1 on the symmetrized skew bidisc

$$ \mathbb {G}_{r} {\mathop {=}\limits ^\textrm{def}} \Big \{( \lambda _{1}+r\lambda _{2} ,r\lambda _{1}\lambda _{2}): \lambda _{1}\in \mathbb {D}, \lambda _{2}\in \mathbb {D}\Big \}, $$

for a fixed $r \in (0,1)$. We show the existence of a realization formula and a model formula for such holomorphic functions.

## Introduction

In this paper we shall generalize some results from long-established function theory of the unit disc $\mathbb {D}$ and from the theory of holomorphic functions on the bidisc $\mathbb {D}^{2}$ and the symmetrized bidisc $\mathbb {G}$ to holomorphic functions on the symmetrized skew bidisc $\mathbb {G}_{r}$, for a fixed $r \in (0,1)$.

Recall that the *Schur class*, $\mathcal {S}(\mathbb {D})$, is the set of holomorphic functions $\varphi$ on the unit disc $\mathbb {D}$ such that the supremum norm $\Vert \varphi \Vert _\infty = \sup _{z\in \mathbb {D}}|\varphi (z)|\le 1$. The notions of models and realizations of functions are useful for the understanding of the Schur class. A *model* of a function $\varphi :\mathbb {D}\rightarrow \mathbb {C}$ is a pair $(\mathcal {M},u)$ where $\mathcal {M}$ is a Hilbert space and *u* is a map from $\mathbb {D}$ to $\mathcal {M}$ such that, for all $\lambda ,\mu \in \mathbb {D}$,

1.1 $$\begin{aligned} 1-\overline{\varphi (\mu )}\varphi (\lambda ) = (1-\bar{\mu }\lambda )\langle u(\lambda ),u(\mu )\rangle _\mathcal {M}, \end{aligned}$$

where $\langle \cdot ,\cdot \rangle _\mathcal {M}$ denotes the inner product in $\mathcal {M}$. A closely related notion is a *realization* of a function $\varphi$ on $\mathbb {D}$, that is, a formula of the form

1.2 $$\begin{aligned} \varphi (\lambda ) =\alpha + \langle \lambda (1-D\lambda )^{-1}\gamma ,\beta \rangle _\mathcal {M}\qquad \text {for all} \ \lambda \in \mathbb {D}, \end{aligned}$$

where $$\begin{bmatrix}\alpha &  1\otimes \beta \\ \gamma \otimes 1 &  D\end{bmatrix}$$ is the matrix of a unitary operator on $\mathbb {C}\oplus \mathcal {M}$.

The connections between models, realizations and the Schur class are revealed in the following theorem.

### Theorem 1.3

Let $\varphi$ be a function on $\mathbb {D}$. The following conditions are equivalent.

(i) $$\varphi \in \mathcal {S}(\mathbb {D})$$;
(ii) $\varphi$ has a model;
(iii) $\varphi$ has a realization.

Proofs of the various implications in this theorem can be found, for instance, in [4]. Models and realizations of functions have proved to be a powerful tool for both operator-theorists (e.g. Nagy and Foias [9]) and control engineers (largely as a tool for computation [8]). In this paper we shall derive versions of model and realization formulae which apply to functions in the “Schur class” of another domain. For a domain $\Omega$ in $\mathbb {C}^n$ the Schur class $\mathcal {S}(\Omega )$ is defined to be the set of holomorphic functions $\varphi$ on $\Omega$ such that the supremum norm $\Vert \varphi \Vert _\infty {\mathop {=}\limits ^\textrm{def}}\sup _{z\in \Omega }|\varphi (z)|$ is at most 1. We are concerned with the domain $\Omega =\mathbb {G}_r$ in $\mathbb {C}^2$, which we now define.

The symmetrized bidisc $\mathbb {G}$ was introduced by Agler and Young in the course of a study of the spectral Nevanlinna-Pick problem for $2 \times 2$ matrix functions, which is a special case of the “$\mu$-synthesis problem” in robust control theory [7]. $\mathbb {G}$ is defined by

1.4 $$\begin{aligned} \mathbb {G}{\mathop {=}\limits ^\textrm{def}} \Big \{( \lambda _{1}+\lambda _{2} ,\lambda _{1}\lambda _{2}): \lambda _{1}\in \mathbb {D}, \lambda _{2}\in \mathbb {D}\Big \}. \end{aligned}$$

It is known that $\mathbb {G}$ is hypoconvex, polynomially convex and starlike about (0, 0), but not convex, see [2, Theorem 2.3]. Here we study a related region in $\mathbb {C}^2$, to wit, the region

$$ \mathbb {G}_{r} = \Big \{( \lambda _{1}+r\lambda _{2} ,r\lambda _{1}\lambda _{2}): \lambda _{1}\in \mathbb {D}, \lambda _{2}\in \mathbb {D}\Big \}, $$

where $0<r<1$. Since $\mathbb {G}_{r}$ is the image of $\mathbb {D}\times r\mathbb {D}$ under the symmetrization map $(z,w) \mapsto (z+w,zw)$, and $\mathbb {D}\times r\mathbb {D}$ is also a bidisk, arguably $\mathbb {G}_{r}$ also deserves the appellation “symmetrized bidisc”. However, this name has become firmly associated with the domain $\mathbb {G}$, and so we propose the nomenclature “symmetrized skew bidisc” for $\mathbb {G}_{r}$, to avoid clashing with established terminology. $\mathbb {G}_{r}$ is also potentially of interest in connection with the spectral Nevanlinna-Pick problem for $2\times 2$-matrix functions. In a personal communication Lukasz Kosinski pointed out that $\mathbb {G}_{r}$ is not pseudoconvex. We shall also have occasion to make use of the domain

1.5 $$\begin{aligned}  &   r \cdot \mathbb {G} {\mathop {=}\limits ^\textrm{def}}\Big \{(r(\lambda _{1}+\lambda _{2}),r^{2}\lambda _{1}\lambda _{2}): \lambda _{1}\in \mathbb {D}, \lambda _{2}\in \mathbb {D}\Big \}\end{aligned}$$

1.6 $$\begin{aligned}  &   =\Big \{(rs,r^2p):(s,p)\in \mathbb {G}\Big \}. \end{aligned}$$

In 2017 Agler and Young [5] derived a realization formula for any function in $\mathcal {S}(\mathbb {G})$ by means of a symmetrization argument. They introduced the following notion:

### Definition 1.7

A $\mathbb {G}$-*model* for a function $\varphi$ on $\mathbb {G}$ is a triple $(\mathcal {M},T,u)$ where $\mathcal {M}$ is a Hilbert space, *T* is a contraction acting on $\mathcal {M}$ and $u:\mathbb {G} \rightarrow \mathcal {M}$ is a holomorphic function such that, for all $s,t\in \mathbb {G}$,

1.8 $$\begin{aligned} 1-\overline{\varphi (t)}\varphi (s)= \langle (1-t_T^* s_T) u(s),u(t)\rangle _\mathcal {M}. \end{aligned}$$

Here, for any point $s=(s_1,s_2)\in \mathbb {G}$ and any contractive linear operator *T* on a Hilbert space $\mathcal {M}$, the operator $s_T$ is defined by

1.9 $$\begin{aligned} s_T=(2s_2T-s_1)(2-s_1T)^{-1}\quad \text{ on } \mathcal {M}. \end{aligned}$$

A *realization* of a function $\varphi$ on $\mathbb {G}$ is a formula of the form

1.10 $$\begin{aligned} \varphi (s) =\alpha + \langle s_T(1-D s_T)^{-1}\gamma ,\beta \rangle _\mathcal {M}\qquad \text {for all} \ s \in \mathbb {G}, \end{aligned}$$

where $$\begin{bmatrix}\alpha &  1\otimes \beta \\ \gamma \otimes 1 &  D\end{bmatrix}$$ is the matrix of a unitary operator on $\mathbb {C}\oplus \mathcal {M}$ and *T* is a contraction on $\mathcal {M}$.

In [5, Theorem 2.2 and Theorem 3.1] Agler and Young proved the following statement.

### Theorem 1.11

Let $\varphi$ be a function on $\mathbb {G}$. The following three statements are equivalent.

1. $$\varphi \in \mathcal {S}(\mathbb {G})$$;
2. $\varphi$ has a $\mathbb {G}$-model $(\mathcal {M}, T, u)$ in which *T* is a unitary operator on $\mathcal {M}$;
3. $$\varphi $$ has a realization.

To study $\mathbb {G}_{r}$, we define the involution $\sigma$ on $\mathbb {C}^{2}$ by

1.12 $$\begin{aligned} \lambda ^{\sigma } = (r\lambda _{2},r^{-1}\lambda _{1})~ \text {for all}~ \lambda = (\lambda _1, \lambda _2) \in \mathbb {C}^{2}. \end{aligned}$$

We perform a symmetrization argument on $\mathbb {D}^2$ using the involution $\sigma$ to obtain a model formula for $\mathbb {G}_{r}$ in Theorem 2.11 and Theorem 3.1. To state the formulae we require the following notation.

### Definition 1.13

Let $r\in (0,1)$, let $\mathcal {M}$ be a complex Hilbert space, let $\mathcal {H}_{1}$ be a closed non-trivial proper subspace of $\mathcal {M}$, and let *U* be a unitary operator on $\mathcal {M}$. We define $\mathcal {R}$ in $\mathcal {B(M)}$ by the formula

1.14 $$\begin{aligned} \mathcal {R}=\begin{bmatrix} 1_{\mathcal {H}_{1} } &  0 \\ 0 &  r\cdot 1_{\mathcal {H}_{1}^{\perp }} \\ \end{bmatrix}\in \mathcal {B(M)}. \end{aligned}$$

For $s=(s_{1},s_{2})\in r\cdot \mathbb {G}$, we define $s_{U,\mathcal {R}}\in \mathcal {B(M)}$ by

1.15 $$\begin{aligned} s_{U,\mathcal {R}} = \bigg (2s_{2}\mathcal {R}^{-1}U-s_{1}\bigg )\bigg (2\mathcal {R}-s_{1}U\bigg )^{-1}. \end{aligned}$$

### Remark 1.16

Let $r\in (0,1)$. The relation between the operator $s_{U,\mathcal {R}}\in \mathcal {B(M)}$ given by equation (1.15) and the operator $s_T\in \mathcal {B(M)}$ given by equation (1.9) is the following. For $s=(s_{1},s_{2})\in r\cdot \mathbb {G}$,

1.17 $$\begin{aligned} s_{U,\mathcal {R}} = s_{\mathcal {R}^{-1}U}\mathcal {R}^{-1}. \end{aligned}$$

Note that $\Vert \mathcal {R}^{-1}U\Vert = r^{-1}$, and so $\mathcal {R}^{-1}U$ is not a contraction, but one can check that, for $s=(s_{1},s_{2})\in r\cdot \mathbb {G}$, the operator $s_{\mathcal {R}^{-1}U}$ is still well defined.

We prove the following results in Lemma 2.32.

### Lemma 1.18

Let $r\in (0,1)$, let $\mathcal {M}$ be a complex Hilbert space, let $\mathcal {H}_{1}$ be a closed non-trivial proper subspace of $\mathcal {M}$, let the operator $\mathcal {R} \in \mathcal {B(M)}$ be defined by equation (1.14) and *U* be a unitary operator on $\mathcal {M}$.

1. The operator-valued function
  $$w:r\cdot \mathbb {G}\rightarrow \mathcal {B(M)} : s\mapsto s_{U,\mathcal {R}},$$
  where $s_{U,\mathcal {R}}\in \mathcal {B(M)}$ is given by equation (1.15), is well defined and holomorphic on $r\cdot \mathbb {G}$;
2. $$\Vert s_{U,\mathcal {R}}\Vert _{\mathcal {B(M)}}< 1$$ for all $$s=(s_{1},s_{2})\in r\cdot \mathbb {G}$$.

### Theorem 1.19

Let $r\in (0,1)$ and let $f\in \mathcal {S}(\mathbb {G}_{r})$. Then there exists a model $(\mathcal {M}, (U,\mathcal {R}), u)$ for *f* on $r\cdot \mathbb {G}$, that is, there exist a complex Hilbert space $\mathcal {M}$, a closed non-trivial proper subspace $\mathcal {H}_{1}$ of $\mathcal {M}$, a holomorphic map $u:r\cdot \mathbb {G}\rightarrow \mathcal {M}$, a unitary operator *U* on $\mathcal {M}$ and the operator $\mathcal {R} \in \mathcal {B(M)}$ given by equation (1.14), such that, for all $s=(s_{1},s_{2})\in r\cdot \mathbb {G}$ and $t=(t_{1},t_{2})\in r \cdot \mathbb {G}$,

1.20 $$\begin{aligned} 1- \overline{f(t)}f(s) =\Bigg \langle \bigg (1_{\mathcal {M}}-t_{U,\mathcal {R}}^{*}s_{U,\mathcal {R}}\bigg )u(s),u(t)\Bigg \rangle _{\mathcal {M}}, \end{aligned}$$

where the operators $s_{U,\mathcal {R}}$ and $t_{U,\mathcal {R}}$ are defined by equation (1.15).

Note that the model formula of a function $f\in \mathcal {S}(\mathbb {G}_{r})$ is similar to the model formula (1.7) of a function $f\in \mathcal {S}(\mathbb {G})$ except that the operators $s_U, t_U$ are replaced by the operators $s_{U,\mathcal {R}}$ and $t_{U,\mathcal {R}}$ respectively, where $R \in \mathcal {B(M)}$ given by equation (1.14).

We prove in Theorem 3.16 a realization formula for functions in $\mathcal {S}(\mathbb {G}_{r})$. Let us state this result.

### Theorem 1.21

Let $r\in (0,1)$ and $f \in \mathcal {S}(\mathbb {G}_{r})$. There exist a scalar $a\in \mathbb {C}$, a complex Hilbert space $\mathcal {M}$, vectors $\beta , \gamma , \in \mathcal {M}$, a closed non-trivial proper subspace $\mathcal {H}_{1}$ of $\mathcal {M}$ and linear operators *D*, *U* on $\mathcal {M}$ such that *D* is a contraction, *U* is unitary such that the operator

1.22 $$\begin{aligned} L=\begin{bmatrix} a &  1\otimes \beta \\ \gamma \otimes 1 &  D \end{bmatrix} \end{aligned}$$

is unitary on $\mathbb {C}\oplus \mathcal {M}$ and, for all $s=(s_{1},s_{2})\in r\cdot \mathbb {G}$,

$$f(s)=a+\langle s_{U,\mathcal {R}}(1-Ds_{U,\mathcal {R}})^{-1}\gamma , \beta \rangle _{\mathcal {M}},$$

where the operator $s_{U,\mathcal {R}}$ is defined by equation (1.15) and the operator $\mathcal {R} \in \mathcal {B(M)}$ given by equation (1.14).

## A Model Formula for the Bidisc $\mathbb {D}^{2}$ and Relations to the Symmetrized Skew Bidisc

As a preliminary to the construction of models of functions on $\mathbb {G}_{r}$, we recall the notion of a Hilbert space model of a function on $\mathbb {D}^{2}$.

### Definition 2.1

[4, Definition 4.18] Let $\varphi$ be a function on $\mathbb {D}^{2}$. A pair $(\mathcal {H},u)$ is said to be a model of $\varphi$ if $\mathcal {H}=\mathcal {H}_{1}\oplus \mathcal {H}_{2}$ is a Hilbert space, $\mathcal {H}_{1}$ and $\mathcal {H}_{2}$ are orthogonally complementary subspaces of $\mathcal {H}$ and $u=(u_{1},u_{2})$ is a pair of holomorphic maps from $\mathbb {D}^{2}$ to $\mathcal {H}_{1},\mathcal {H}_{2}$ respectively such that, for all $\lambda =(\lambda _1, \lambda _2), \ \mu = (\mu _1, \mu _2) \in \mathbb {D}^{2}$,

2.2 $$\begin{aligned} 1-\overline{\varphi (\mu )}\varphi (\lambda ) = \big \langle (1-\overline{\mu _{1}}\lambda _{1}) u_{1}({\lambda }),u_{1}({\mu })\big \rangle _{\mathcal {H}_{1}}+\big \langle (1-\overline{\mu _{2}}\lambda _{2}) u_{2}({\lambda }),u_{2}(\mu )\big \rangle _{\mathcal {H}_{2}}. \end{aligned}$$

It was proved by Agler in [1] that any holomorphic function $\varphi : \mathbb {D}^{2} \rightarrow \overline{\mathbb {D}}$ has a model.

### Theorem 2.3

(Agler) A function $\varphi$ on $\mathbb {D}^{2}$ belongs to the Schur class $\mathcal {S}(\mathbb {D}^{2})$ if and only if $\varphi$ has a model.

To study $\mathbb {G}_{r}$, we define the involution $\sigma$ on $\mathbb {C}^{2}$ by

2.4 $$\begin{aligned} \lambda ^{\sigma } = (r\lambda _{2},r^{-1}\lambda _{1})~ \text {for all}~ \lambda = (\lambda _1, \lambda _2) \in \mathbb {C}^{2}. \end{aligned}$$

Note that, for all $\lambda \in r\mathbb {D}\times \mathbb {D}$, we have $\lambda ^{\sigma }\in r\mathbb {D}\times \mathbb {D}$ and

2.5 $$\begin{aligned} (\lambda ^{\sigma })^{\sigma } = (r\lambda _{2},r^{-1}\lambda _{1})^{\sigma } = (rr^{-1}\lambda _{1},r^{-1}r\lambda _{2}) = \lambda . \end{aligned}$$

This implies $(r\mathbb {D}\times \mathbb {D})^{\sigma } = r\mathbb {D}\times \mathbb {D}.$ Define the operator $T_{r}:\mathbb {C}^{2} \rightarrow \mathbb {C}^{2}$ by

2.6 $$\begin{aligned} T_{r}(\lambda _{1},\lambda _{2})= (\lambda _{1},r\lambda _{2}) \ \text {for} \ \lambda =(\lambda _{1},\lambda _{2})\in \mathbb {C}^{2}. \end{aligned}$$

Define also the map $\pi : \mathbb {C}^{2} \rightarrow \mathbb {C}^{2}$ by the formula

2.7 $$\begin{aligned} \pi (\lambda _{1},\lambda _{2}) = (\lambda _{1}+\lambda _{2}, \lambda _{1}\lambda _{2}) \ \text {for} \ (\lambda _1, \lambda _2) \in \mathbb {C}^{2}, \end{aligned}$$

so that we have $\mathbb {G}_{r} = \pi (\mathbb {D}\times r\mathbb {D})$. Note that, for $\lambda = (r\lambda _{1},\lambda _{2}) \in r\mathbb {D}\times \mathbb {D}$, $\lambda ^{\sigma }=(r\lambda _{2},\lambda _{1})$ and

$$\begin{aligned}&\pi \big (T_{r}(\lambda )\big ) = \pi (r\lambda _{1},r\lambda _{2}) = (r(\lambda _{1}+\lambda _{2}),r^{2}\lambda _{1}\lambda _{2}),\\&\pi \big (T_{r}(\lambda ^{\sigma })\big ) = \pi \big (T_{r}(r\lambda _{2},\lambda _{1})\big ) = \pi (r\lambda _{2},r\lambda _{1})= (r(\lambda _{1}+\lambda _{2}),r^{2}\lambda _{1}\lambda _{2}). \end{aligned}$$

Thus, for all $\lambda \in r\mathbb {D}\times \mathbb {D}$,

2.8 $$\begin{aligned} \pi \big (T_{r}(\lambda )\big ) = \pi \big (T_{r}(\lambda ^{\sigma })\big ). \end{aligned}$$

Let $f : \mathbb {G}_{r} \rightarrow \overline{\mathbb {D}}$ be a holomorphic function. Then we may define $F:\mathbb {D}^{2}\rightarrow \overline{\mathbb {D}}$ by

2.9 $$\begin{aligned} F = f \circ \pi \circ T_{r} : \mathbb {D}^{2} \rightarrow \overline{\mathbb {D}}. \end{aligned}$$

It is clear that *F* is in the Schur class of $\mathbb {D}^{2}$. Note that, by equation (2.8), *F* is symmetric with respect to the involution $\sigma$,

2.10 $$\begin{aligned} F(\lambda ^{\sigma }) = f(\pi (r\lambda _{2},\lambda _{1})) = f(\lambda _{1}+r\lambda _{2},r\lambda _{1}\lambda _{2}) = F(\lambda ), \ \text {for all} \ \lambda \in r\mathbb {D}\times \mathbb {D}. \end{aligned}$$

We now bring all these notions together with the model of a function on $\mathbb {D}^{2}$ to prove the following statement.

### Theorem 2.11

Let $f\in \textrm{Hol}(\mathbb {G}_{r},\overline{\mathbb {D}})$ and let

$$F = f \circ \pi \circ T_{r}: \mathbb {D}^{2} \rightarrow \overline{\mathbb {D}}.$$

Then there exist a complex Hilbert space $\mathcal {M}$, a closed non-trivial proper subspace $\mathcal {H}_{1}$ of $\mathcal {M}$, a unitary operator *U* on $\mathcal {M}$, a holomorphic map $w: r\mathbb {D}\times \mathbb {D} \rightarrow \mathcal {M}$, which satisfies $w(\lambda ^{\sigma }) = w(\lambda )$ for all $\lambda \in r\mathbb {D}\times \mathbb {D}$, such that, for all $\lambda , \mu \in r\mathbb {D}\times \mathbb {D}$,

2.12 $$\begin{aligned} 1-\overline{F(\mu )}F(\lambda ) = \langle Z_{r}(\lambda ,\mu )w(\lambda ),w(\mu ) \rangle _{\mathcal {M}}, \end{aligned}$$

where

$$\begin{aligned} Z_{r}(\lambda ,\mu ) =&(1_{\mathcal {M}}-r\overline{\mu _{2}}\mathcal {R}^{-1}U^{*})(1_{\mathcal {M}}- \overline{\mu }_{1}\lambda _{1}\mathcal {R}^{-2})(1_{\mathcal {M}}-r\lambda _{2}U\mathcal {R}^{-1})\\ +&(1_{\mathcal {M}}-\overline{\mu _{1}}\mathcal {R}^{-1}U^{*})(1_{\mathcal {M}}-r^{2} \overline{\mu }_{2}\lambda _{2}\mathcal {R}^{-2})(1_{\mathcal {M}}-\lambda _{1}U\mathcal {R}^{-1}) \end{aligned}$$

and $\mathcal {R}\in \mathcal {B(M)}$ is defined by equation (1.14).

### Proof

Since $F \in \mathcal {S}(\mathbb {D}^{2})$, by Agler’s Theorem 2.3, *F* has a model $(\mathcal {H},u)$, that is, there exists an orthogonally decomposed Hilbert space $\mathcal {H}=\mathcal {H}_{1}\oplus \mathcal {H}_{2}$ and a pair of holomorphic maps $u=(u_{1},u_{2})$ from $\mathbb {D}^{2}$ to $\mathcal {H}_{1},\mathcal {H}_{2}$ respectively such that, for all $\lambda , \mu \in \mathbb {D}^{2}$,

2.13 $$\begin{aligned} 1-\overline{F(\mu )}{F(\lambda )}=\langle (1-\overline{\mu _{1}}\lambda _{1}) u_{1}({\lambda }),u_{1}({\mu })\rangle _{\mathcal {H}_{1}}+\langle (1-\overline{\mu }_{2}\lambda _{2}) u_{2}({\lambda }),u_{2}({\mu })\rangle _{\mathcal {H}_{2}}. \end{aligned}$$

Consider $\lambda$ and $\mu$ in $r\mathbb {D}\times \mathbb {D}$, replace $\lambda , \mu$ by $\lambda ^{\sigma }, \mu ^{\sigma }$ respectively in equation (2.13) and use equation (2.10) to deduce that, for all $\lambda$ and $\mu$ in $r\mathbb {D}\times \mathbb {D}$, the following equation holds

2.14 $$\begin{aligned} 1- &   {\overline{F(\mu )}}{F(\lambda )} \nonumber \\= &   (1-r^{2}\overline{\mu _{2}}\lambda _{2})\langle {u_{1}({\lambda ^{\sigma }}),u_{1}({\mu }^{\sigma }) \rangle _{\mathcal {H}_{1}}}+\langle {(1-r^{-2}\overline{\mu _{1}}\lambda _{1})u_{2}({\lambda ^{\sigma }}), u_{2}({\mu ^{\sigma }})\rangle _{\mathcal {H}_{2}}}. \end{aligned}$$

Take the average of equations (2.13) and (2.14) to obtain, for all $\lambda$ and $\mu$ in $r\mathbb {D}\times \mathbb {D}$,

$$\begin{aligned} 1-&\overline{F(\mu )}F(\lambda ) \\ =&\dfrac{1}{2}\Bigg (\Big \langle (1-\overline{\mu }_{1}\lambda _{1})u_{1}({\lambda }),u_{1}(\mu )\Big \rangle _{\mathcal {H}_{1}}+ \Big \langle (1-r^{-2}\overline{\mu }_{1}\lambda _{1})u_{2}({\lambda ^{\sigma }}), u_{2}(\mu ^{\sigma })\Big \rangle _{\mathcal {H}_{2}} \\&+ \Big \langle (1-r^{2}\overline{\mu }_{2}\lambda _{2}) u_{1}(\lambda ^{\sigma }), u_{1}(\mu ^{\sigma })\Big \rangle _{\mathcal {H}_{1}}+\Big \langle (1-\overline{\mu }_{2}\lambda _{2})u_{2}(\lambda ), u_{2}(\mu )\Big \rangle _{\mathcal {H}_{2}}\Bigg ). \end{aligned}$$

The last equation can be be re-written as

2.15 $$\begin{aligned} 1-\overline{F(\mu )}F(\lambda )= &   \dfrac{1}{2}\Bigg (\Bigg \langle \begin{bmatrix} (1-\overline{\mu }_{1}\lambda _{1})u_{1}(\lambda ) \\ (1-r^{-2}\overline{\mu }_{1}\lambda _{1})u_{2}(\lambda ^{\sigma }) \\ \end{bmatrix}, \begin{bmatrix} u_{1}(\mu ) \\ u_{2}(\mu ^{\sigma }) \\ \end{bmatrix}\Bigg \rangle _{\mathcal {H}_{1}\oplus \mathcal {H}_{2}}\nonumber \\  &   + \Bigg \langle \begin{bmatrix} (1-r^{2}\overline{\mu }_{2}\lambda _{2})u_{1}({\lambda ^{\sigma }}) \\ (1-\overline{\mu }_{2}\lambda _{2})u_{2}({\lambda }) \\ \end{bmatrix}, \begin{bmatrix} u_{1}({\mu }^{\sigma }) \\ u_{2}({\mu }) \\ \end{bmatrix}\Bigg \rangle _{\mathcal {H}_{1}\oplus \mathcal {H}_{2}}\Bigg ). \end{aligned}$$

For each $\lambda \in r\mathbb {D}\times \mathbb {D}$, define the vector $v(\lambda )\in \mathcal {H}$ and the operator $\widetilde{\mathcal {R}} \in \mathcal {B(H)}$ by

$$\begin{aligned} v({\lambda }) = \dfrac{1}{\sqrt{2}} \begin{bmatrix} u_{1}({\lambda }) \\ u_{2}({\lambda ^{\sigma }}) \\ \end{bmatrix},~ \widetilde{\mathcal {R}} = \begin{bmatrix} 1_{\mathcal {H}_1} &  0 \\ 0 &  r\cdot 1_{\mathcal {H}_2} \\ \end{bmatrix}. \end{aligned}$$

Then, for all $\lambda ,\mu \in r\mathbb {D}\times \mathbb {D}$, equation (2.15) can be written as

2.16 $$\begin{aligned} 1-\overline{F(\mu )}F(\lambda )&= \Big \langle (1_{\mathcal {H}}-\overline{\mu }_{1}\lambda _{1}\widetilde{\mathcal {R}}^{-2})v({\lambda }), v({\mu })\Big \rangle _{\mathcal {H}}\nonumber \\&\quad +\Big \langle (1_{\mathcal {H}}-r^{2}\overline{\mu }_{2}\lambda _{2}\widetilde{\mathcal {R}}^{-2}) v({\lambda }^{\sigma }),v(\mu ^{\sigma })\Big \rangle _{\mathcal {H}}. \end{aligned}$$

Again, use the fact that $F(\lambda ^{\sigma })=F(\lambda )$ for all $\lambda \in r \mathbb {D}\times \mathbb {D}$ and replace $\lambda$ with $\lambda ^{\sigma }$ in equation (2.16) to obtain

2.17 $$\begin{aligned} 1-\overline{F(\mu )}F(\lambda )&= \Big \langle (1_{\mathcal {H}}-r\overline{\mu }_{1}\lambda _{2}\widetilde{\mathcal {R}}^{-2})v(\lambda ^{\sigma }),v(\mu )\Big \rangle _{\mathcal {H}}\end{aligned}$$

2.18 $$\begin{aligned}&\quad +\Big \langle (1_{\mathcal {H}}-r\overline{\mu }_{2}\lambda _{1}\widetilde{\mathcal {R}}^{-2})v({\lambda }),v({\mu }^{\sigma })\Big \rangle _{\mathcal {H}}. \end{aligned}$$

We then equate the right hand sides of equations (2.16) and (2.17) to see that

$$\begin{aligned} \Big \langle (1_{\mathcal {H}}-\overline{\mu }_{1}&\lambda _{1}\widetilde{\mathcal {R}}^{-2})v({\lambda }), v({\mu })\Big \rangle _{\mathcal {H}}+\Big \langle (1_{\mathcal {H}}-r^{2}\overline{\mu }_{2}\lambda _{2} \widetilde{\mathcal {R}}^{-2})v({\lambda }^{\sigma }),v({\mu }^{\sigma })\Big \rangle _{\mathcal {H}} \\ = \Big \langle (1_{\mathcal {H}}-&r\overline{\mu }_{1}\lambda _{2}\widetilde{\mathcal {R}}^{-2})v(\lambda ^{\sigma }), v(\mu )\Big \rangle _{\mathcal {H}}+\Big \langle (1_{\mathcal {H}}-r\overline{\mu }_{2}\lambda _{1} \widetilde{\mathcal {R}}^{-2})v({\lambda }),v({\mu }^{\sigma })\Big \rangle _{\mathcal {H}}. \end{aligned}$$

Expanding brackets, we find that

2.19 $$\begin{aligned}  &   \Big \langle v(\lambda ), v(\mu ) \Big \rangle _{\mathcal {H}} - \Big \langle \overline{\mu }_{1}\lambda _{1} \widetilde{\mathcal {R}}^{-2} v(\lambda ), v(\mu )\Big \rangle _{\mathcal {H}} \nonumber \\  &   \qquad + \Big \langle v(\lambda ^{\sigma }), v(\mu ^{\sigma })\Big \rangle _{\mathcal {H}} - \Big \langle r^{2}\overline{\mu }_{2}\lambda _{2}\widetilde{\mathcal {R}}^{-2}v(\lambda ^{\sigma }), v(\mu ^{\sigma }) \Big \rangle _{\mathcal {H}} \nonumber \\  &   \quad =\Big \langle v(\lambda ^{\sigma }), v(\mu ) \Big \rangle _{\mathcal {H}} - \Big \langle r\overline{\mu }_{1}\lambda _{2}\widetilde{\mathcal {R}}^{-2}v(\lambda ^{\sigma }), v(\mu ) \Big \rangle _{\mathcal {H}}\nonumber \\  &   \qquad + \Big \langle v(\lambda ), v(\mu ^{\sigma })\Big \rangle _{\mathcal {H}}-\Big \langle r\overline{\mu }_{2}\lambda _{1}\widetilde{\mathcal {R}}^{-2}v(\lambda ),v(\mu ^{\sigma })\Big \rangle _{\mathcal {H}} . \end{aligned}$$

Rearrange equation (2.19) to obtain, for all $\lambda ,\mu \in r\mathbb {D}\times \mathbb {D}$,

$$\begin{aligned} \Big \langle v(\lambda ), v(\mu ) \Big \rangle _{\mathcal {H}}&+ \Big \langle v(\lambda ^{\sigma }),v(\mu ^{\sigma })\Big \rangle _{\mathcal {H}} - \Big \langle v(\lambda ^{\sigma }), v(\mu )\Big \rangle _{\mathcal {H}} - \Big \langle v(\lambda ), v(\mu ^{\sigma })\Big \rangle _{\mathcal {H}} \\&=\Big \langle \overline{\mu }_{1}\lambda _{1} \widetilde{\mathcal {R}}^{-2} v(\lambda ), v(\mu )\Big \rangle _{\mathcal {H}} +\Big \langle r^{2}\overline{\mu }_{2}\lambda _{2}\widetilde{\mathcal {R}}^{-2}v(\lambda ^{\sigma }), v(\mu ^{\sigma }) \Big \rangle _{\mathcal {H}} \\&\qquad - \Big \langle r\overline{\mu }_{1}\lambda _{2}\widetilde{\mathcal {R}}^{-2}v(\lambda ^{\sigma }), v(\mu ) \Big \rangle _{\mathcal {H}} -\Big \langle r\overline{\mu }_{2}\lambda _{1}\widetilde{\mathcal {R}}^{-2}v(\lambda ),v(\mu ^{\sigma })\Big \rangle _{\mathcal {H}}. \end{aligned}$$

The last equation can be simplified to

$$\begin{aligned} \Big \langle v(\lambda ) - v(\lambda ^{\sigma }),&\ v(\mu )\Big \rangle _{\mathcal {H}} + \Big \langle v(\lambda ^{\sigma })-v(\lambda ), v(\mu ^{\sigma })\Big \rangle _{\mathcal {H}} \\&=\Big \langle \overline{\mu }_{1}\lambda _{1} \widetilde{\mathcal {R}}^{-2} v(\lambda )-r\overline{\mu }_{1}\lambda _{2}\widetilde{\mathcal {R}}^{-2}v(\lambda ^{\sigma }), v(\mu )\Big \rangle _{\mathcal {H}} \\&\qquad + \Big \langle r^{2}\overline{\mu }_{2}\lambda _{2}\widetilde{\mathcal {R}}^{-2}v(\lambda ^{\sigma }) - r\overline{\mu }_{2}\lambda _{1}\widetilde{\mathcal {R}}^{-2}v(\lambda ),v(\mu ^{\sigma }) \Big \rangle _{\mathcal {H}} \end{aligned}$$

and then to

2.20 $$\begin{aligned}  &   \Big \langle v(\lambda ) - v(\lambda ^{\sigma }), v(\mu )-v(\mu ^{\sigma })\Big \rangle _{\mathcal {H}} \nonumber \\  &   \quad =\Big \langle \overline{\mu }_{1}\widetilde{\mathcal {R}}^{-2}\big (\lambda _{1} v(\lambda )-r\lambda _{2}v(\lambda ^{\sigma })\big ), v(\mu )\Big \rangle _{\mathcal {H}} \nonumber \\  &   \qquad +\Big \langle r\overline{\mu }_{2}\widetilde{\mathcal {R}}^{-2}\big (r\lambda _{2}v(\lambda ^{\sigma }) - \lambda _{1}v(\lambda )\big ),v(\mu ^{\sigma }) \Big \rangle _{\mathcal {H}} . \end{aligned}$$

The equation (2.20) can then be written in the form

$$\begin{aligned}&\Big \langle v(\lambda ) - v(\lambda ^{\sigma }), v(\mu )-v(\mu ^{\sigma })\Big \rangle _{\mathcal {H}} \\&\quad =\Big \langle \widetilde{\mathcal {R}}^{-1}\big (\lambda _{1} v(\lambda )-r\lambda _{2}v(\lambda ^{\sigma })\big ), \mu _{1}\widetilde{\mathcal {R}}^{-1}v(\mu )\Big \rangle _{\mathcal {H}} \\&\qquad +\Big \langle \widetilde{\mathcal {R}}^{-1}\big (r\lambda _{2}v(\lambda ^{\sigma }) - \lambda _{1}v(\lambda )\big ),r\mu _{2}\widetilde{\mathcal {R}}^{-1}v(\mu ^{\sigma }) \Big \rangle _{\mathcal {H}} \end{aligned}$$

and further simplified to the form

$$\begin{aligned}&\Big \langle v(\lambda ) - v(\lambda ^{\sigma }), v(\mu )-v(\mu ^{\sigma })\Big \rangle _{\mathcal {H}} \\&\quad =\Big \langle \widetilde{\mathcal {R}}^{-1}\big (\lambda _{1} v(\lambda )-r\lambda _{2}v(\lambda ^{\sigma })\big ), \widetilde{\mathcal {R}}^{-1}\big (\mu _{1}v(\mu )-r\mu _{2}v(\mu ^{\sigma })\big )\Big \rangle _{\mathcal {H}}. \end{aligned}$$

This is equivalent to saying that the Gramian of the family

$$\{v({\lambda })-v({\lambda }^{\sigma }):\lambda \in r\mathbb {D}\times \mathbb {D}\}$$

in $\mathcal {H}$ is equal to the Gramian of the family

$$\{\widetilde{\mathcal {R}}^{-1}(\lambda _{1}v({\lambda })-r\lambda _{2}v({\lambda }^{\sigma })) : \lambda \in r\mathbb {D}\times \mathbb {D}\},$$

also in $\mathcal {H}$. Hence there exists a linear isometry

$$\begin{aligned} L : \overline{{{\,\textrm{Span}\,}}}\Big \{\widetilde{\mathcal {R}}^{-1}(\lambda _{1}v({\lambda })- r\lambda _{2}v({\lambda }^{\sigma }))&: \lambda \in r\mathbb {D}\times \mathbb {D}\Big \} \\&\rightarrow \overline{{{\,\textrm{Span}\,}}}\Big \{v({\lambda })-v({\lambda }^{\sigma }) : \lambda \in r\mathbb {D}\times \mathbb {D}\Big \} \end{aligned}$$

with

2.21 $$\begin{aligned} L\Big ( \widetilde{\mathcal {R}}^{-1}(\lambda _{1}v({\lambda })-r\lambda _{2}v({\lambda }^{\sigma })\Big ) = v({\lambda })-v({\lambda }^{\sigma }), \end{aligned}$$

for all $\lambda \in r\mathbb {D}\times \mathbb {D}$. For subsequent calculations, it becomes advantageous to extend *L* to a unitary operator *U* on a Hilbert space $\mathcal {M}\supseteq \mathcal {H}$. We also extend $\widetilde{\mathcal {R}}$ to an operator $\mathcal {R}$ on the Hilbert space $\mathcal {M}=\mathcal {H}_{1}\oplus \mathcal {H}_{1}^{\perp }$, where $\mathcal {H}_{1}^{\perp }= \mathcal {M} \ominus \mathcal {H}_{1}$, by

$$\begin{aligned} \mathcal {R} = \begin{bmatrix} \widetilde{\mathcal {R}}_{\mathcal {H}} &  0 \\ 0 &  r_{\mathcal {H}^{\perp }} \\ \end{bmatrix} = \begin{bmatrix} 1_{\mathcal {H}_{1}} &  0 &  0 \\ 0 &  r\cdot 1_{\mathcal {H}_{2}} &  0 \\ 0 &  0 &  r\cdot 1_{\mathcal {H}^{\perp }} \\ \end{bmatrix} = \begin{bmatrix} 1_{\mathcal {H}_{1} } &  0 \\ 0 &  r\cdot 1_{\mathcal {H}_{1}^{\perp }} \\ \end{bmatrix}. \end{aligned}$$

We rearrange equation (2.21) with *L* replaced by *U* to obtain

2.22 $$\begin{aligned} (1_{\mathcal {M}}-\lambda _{1}U\mathcal {R}^{-1})v({\lambda }) = (1_{\mathcal {M}}-r\lambda _{2}U\mathcal {R}^{-1})v({\lambda }^{\sigma }), \end{aligned}$$

for all $\lambda \in r\mathbb {D}\times \mathbb {D}$. Since $\mathcal {R}$ is a diagonal operator on $\mathcal {M}$ and $\lambda _{1}\in r\mathbb {D}$, we obtain

$$\Vert \lambda _{1}U\mathcal {R}^{-1}\Vert _{\mathcal {B(M)}} = |\lambda _{1} |\Vert \mathcal {R}^{-1} \Vert _{\mathcal {B(M)}} = \dfrac{|\lambda _{1} |}{r} < 1. $$

Hence $1_{\mathcal {M}}-\lambda _{1}U\mathcal {R}^{-1}$ is invertible. Likewise, since

$$ \Vert r \lambda _{2} U\mathcal {R}^{-1}\Vert _{\mathcal {B(M)}} =r |\lambda _{2}|\Vert \mathcal {R}^{-1}\Vert _{\mathcal {B(M)}} = |\lambda _{2} |< 1,$$

the operator $1_{\mathcal {M}}-r\lambda _{2}U\mathcal {R}^{-1}$ is also invertible. Note that

2.23 $$\begin{aligned} (1_{\mathcal {M}}-r\lambda _{2}U\mathcal {R}^{-1})(1_{\mathcal {M}}-\lambda _{1}U\mathcal {R}^{-1}) = (1_{\mathcal {M}}-\lambda _{1}U\mathcal {R}^{-1})(1_{\mathcal {M}}-r\lambda _{2}U\mathcal {R}^{-1}), \end{aligned}$$

which can be verified by expanding brackets. Multiply both sides of equation (2.23) on the left and right by $(1_{\mathcal {M}}-\lambda _{1}U\mathcal {R}^{-1})^{-1}$ to produce

2.24 $$\begin{aligned} (1_{\mathcal {M}}-\lambda _{1}U\mathcal {R}^{-1})^{-1}(1_{\mathcal {M}}-r\lambda _{2}U\mathcal {R}^{-1}) = (1_{\mathcal {M}}-r\lambda _{2}U\mathcal {R}^{-1})(1_{\mathcal {M}}-\lambda _{1}U\mathcal {R}^{-1})^{-1}. \end{aligned}$$

Rearrange equation (2.22) to

2.25 $$\begin{aligned} v({\lambda }) = (1_{\mathcal {M}}-\lambda _{1}U\mathcal {R}^{-1})^{-1}(1_{\mathcal {M}}-r\lambda _{2}U\mathcal {R}^{-1})v({\lambda }^{\sigma }), \end{aligned}$$

for all $\lambda \in r\mathbb {D}\times \mathbb {D}$. By equation (2.24), equation (2.25) can be written

2.26 $$\begin{aligned} v({\lambda }) = (1_{\mathcal {M}}-r\lambda _{2}U\mathcal {R}^{-1})(1_{\mathcal {M}}-\lambda _{1}U\mathcal {R}^{-1})^{-1}v({\lambda }^{\sigma }). \end{aligned}$$

Thus

2.27 $$\begin{aligned} (1_{\mathcal {M}}-r\lambda _{2}U\mathcal {R}^{-1})^{-1}v({\lambda }) = (1_{\mathcal {M}}-\lambda _{1}U\mathcal {R}^{-1})^{-1}v({\lambda }^{\sigma }), \end{aligned}$$

for all $\lambda \in r\mathbb {D}\times \mathbb {D}$. Let us define $w: r\mathbb {D}\times \mathbb {D}\rightarrow \mathcal {M}$ by

2.28 $$\begin{aligned} w(\lambda )=(1_{\mathcal {M}}-r\lambda _{2}U\mathcal {R}^{-1})^{-1}v({\lambda })~~\text {for all}~\lambda \in r\mathbb {D}\times \mathbb {D}. \end{aligned}$$

Note, for $$\lambda \in r \mathbb {D}\times \mathbb {D}$$,

2.29 $$\begin{aligned} v({\lambda })= &   (1_{\mathcal {M}}-r\lambda _{2}U\mathcal {R}^{-1})w(\lambda ) ,\end{aligned}$$

2.30 $$\begin{aligned} ~v({\lambda }^{\sigma })= &   (1_{\mathcal {M}}-\lambda _{1}U\mathcal {R}^{-1})w(\lambda ^{\sigma }) . \end{aligned}$$

Thus, by equation (2.27), for $\lambda \in r \mathbb {D} \times \mathbb {D}$,

2.31 $$\begin{aligned} w({\lambda ^{\sigma }})= &   (1_{\mathcal {M}}-\lambda _{1}U\mathcal {R}^{-1})^{-1}v({\lambda }^{\sigma }) \nonumber \\= &   (1_{\mathcal {M}}-r\lambda _{2}U\mathcal {R}^{-1})^{-1}v({\lambda }) \nonumber \\= &   w(\lambda ). \end{aligned}$$

Hence *w* is symmetric with respect to the involution $\sigma$ on $r\mathbb {D}\times \mathbb {D}$. Substituting the expressions (2.29) and (2.30) into equation (2.16) and enlarging $\mathcal {H}$ to $\mathcal {M}$, we find that, for all $\lambda \in r\mathbb {D}\times \mathbb {D}$,

$$\begin{aligned} 1-&\overline{F(\mu )}F(\lambda ) \\ =&\Big \langle (1_{\mathcal {M}}-\overline{\mu }_{1}\lambda _{1}\mathcal {R}^{-2})(1_{\mathcal {M}}-r\lambda _{2}U\mathcal {R}^{-1}) w(\lambda ),(1_{\mathcal {M}}-r\mu _{2}U\mathcal {R}^{-1})w(\mu )\Big \rangle _{\mathcal {M}} \\&+\Big \langle (1_{\mathcal {M}}-r^{2} \overline{\mu }_{2}\lambda _{2}\mathcal {R}^{-2})(1_{\mathcal {M}}-\lambda _{1}U\mathcal {R}^{-1}) w(\lambda ), (1_{\mathcal {M}}-\mu _{1}U\mathcal {R}^{-1}) w(\mu ) \Big \rangle _{\mathcal {M}}\\ =&\Big \langle (1_{\mathcal {M}}-r\mu _{2}U\mathcal {R}^{-1})^{*}(1_{\mathcal {M}}-\overline{\mu }_{1}\lambda _{1}\mathcal {R}^{-2})(1_{\mathcal {M}}-r\lambda _{2}U\mathcal {R}^{-1}) w(\lambda ),w(\mu )\Big \rangle _{\mathcal {M}} \\&+\Big \langle (1_{\mathcal {M}}-\mu _{1}U\mathcal {R}^{-1})^{*}(1_{\mathcal {M}}-r^{2} \overline{\mu }_{2}\lambda _{2}\mathcal {R}^{-2})(1_{\mathcal {M}}-\lambda _{1}U\mathcal {R}^{-1}) w(\lambda ), w(\mu ) \Big \rangle _{\mathcal {M}}\\ =&\Big \langle (1_{\mathcal {M}}-r\overline{\mu _{2}}\mathcal {R}^{-1}U^{*})(1_{\mathcal {M}}-\overline{\mu }_{1}\lambda _{1}\mathcal {R}^{-2})(1_{\mathcal {M}}-r\lambda _{2}U\mathcal {R}^{-1})w(\lambda ),w(\mu )\Big \rangle _{\mathcal {M}} \\&+\Big \langle (1_{\mathcal {M}}-\overline{\mu _{1}}\mathcal {R}^{-1}U^{*})(1_{\mathcal {M}}-r^{2} \overline{\mu }_{2}\lambda _{2}\mathcal {R}^{-2})(1_{\mathcal {M}}-\lambda _{1}U\mathcal {R}^{-1}) w(\lambda ), w(\mu ) \Big \rangle _{\mathcal {M}}. \end{aligned}$$

Thus, for all $\lambda \in r\mathbb {D}\times \mathbb {D}$,

$$ 1-\overline{F(\mu )}F(\lambda ) = \langle Z_{r}(\lambda ,\mu ) w(\lambda ),w(\mu )\rangle _{\mathcal {M}}, $$

where

$$\begin{aligned} Z_{r}(\lambda ,\mu ) =&(1_{\mathcal {M}}-r\overline{\mu _{2}}\mathcal {R}^{-1}U^{*})(1_{\mathcal {M}}-\overline{\mu }_{1}\lambda _{1}\mathcal {R}^{-2})(1_{\mathcal {M}}- r\lambda _{2}U\mathcal {R}^{-1}) \\&+ (1_{\mathcal {M}}-\overline{\mu _{1}}\mathcal {R}^{-1}U^{*})(1_{\mathcal {M}}-r^{2} \overline{\mu }_{2}\lambda _{2}\mathcal {R}^{-2})(1_{\mathcal {M}}-\lambda _{1}U\mathcal {R}^{-1}). \end{aligned}$$

Therefore equation (2.12) holds. $\square$

Observe that the domain $r\cdot \mathbb {G}$ defined in equation (1.51.6) can be expressed in terms of the symmetrization map $\pi$ by

$$ r \cdot \mathbb {G}:=\pi (r\mathbb {D}\times r\mathbb {D}).$$

### Lemma 2.32

Let $r\in (0,1)$, let $\mathcal {M}$ be a complex Hilbert space, let $\mathcal {H}_{1}$ be a closed non-trivial proper subspace of $\mathcal {M}$, let

2.33 $$\begin{aligned} \mathcal {R}=\begin{bmatrix} 1_{\mathcal {H}_{1} } &  0 \\ 0 &  r\cdot 1_{\mathcal {H}_{1}^{\perp }} \\ \end{bmatrix}\in \mathcal {B(M)}, \end{aligned}$$

let *D* be a contraction on $\mathcal {M}$ and let *U* be a unitary operator on $\mathcal {M}$.

1. The operator-valued function
  $$w:r\cdot \mathbb {G}\rightarrow \mathcal {B(M)} : s\mapsto s_{U,\mathcal {R}},$$
  where, for $$s=(s_{1},s_{2})\in r\cdot \mathbb {G}$$,
  2.34 $$\begin{aligned} s_{U,\mathcal {R}} = \bigg (2s_{2}\mathcal {R}^{-1}U-s_{1}\bigg )\bigg (2\mathcal {R}-s_{1}U\bigg )^{-1}, \end{aligned}$$
  is well defined and holomorphic on $r\cdot \mathbb {G}$;
2. $$\Vert s_{U,\mathcal {R}}\Vert _{\mathcal {B(M)}}< 1$$ for all $$s=(s_{1},s_{2})\in r\cdot \mathbb {G}$$;
3. for every $\gamma \in \mathcal {M}$, the $\mathcal {M}$-valued function
  $$u:r\cdot \mathbb {G}\rightarrow \mathcal {M} \ \text { defined by} \ u(s) = (1_{\mathcal {M}}-Ds_{U,\mathcal {R}})^{-1}\gamma $$
  is holomorphic on $r\cdot \mathbb {G}$.

### Proof

(1). Let us first check that the definition (2.34) is valid. Since $\mathcal {R}$ is invertible,

$$\bigg (2\mathcal {R}-s_{1}U\bigg ) = \bigg (1_{\mathcal {M}}-\dfrac{1}{2}s_{1}U\mathcal {R}^{-1}\bigg )\bigg (2\mathcal {R}\bigg ) .$$

Note that the operator

$$1_{\mathcal {M}}-\dfrac{1}{2}s_{1}U\mathcal {R}^{-1}$$

is invertible in $\mathcal {B(M)}$ for all $s=(s_{1},s_{2})\in r\cdot \mathbb {G}$. Indeed, for $s_{1}=r \lambda _{1}+r\lambda _{2}$ such that $\lambda _{1}\in \mathbb {D}$ and $\lambda _{2}\in \mathbb {D}$,

$$ \bigg \Vert \dfrac{1}{2} s_{1}U\mathcal {R}^{-1}\bigg \Vert _{\mathcal {B(\mathcal {M})}} = \dfrac{1}{2} |s_{1} |\Vert \mathcal {R}^{-1} \Vert _{\mathcal {B(\mathcal {M})}} < \dfrac{1}{2r}\big (2r\big ) = 1,$$

therefore the inverse of $1_{\mathcal {M}}-\dfrac{1}{2}s_{1}U\mathcal {R}^{-1}$ exists. Hence, $\bigg (2\mathcal {R}-s_{1}U\bigg )$ is also invertible in $\mathcal {B(M)}$ for all $s=(s_{1},s_{2})\in r\cdot \mathbb {G}$. By [6, Proposition I.2.6], for any $T\in \mathcal {B(M)}$, the map

$$g:\textrm{Inv}(\mathcal {B(M)}) \rightarrow \textrm{Inv}(\mathcal {B(M)}),$$

given by $g:T\mapsto T^{-1}$ is holomorphic on $\textrm{Inv}(\mathcal {B(M))}$. Therefore, the operator-valued function

$$w:r\cdot \mathbb {G}\rightarrow \mathcal {B(M)} : s \mapsto s_{U,\mathcal {R}},$$

where $s_{U,\mathcal {R}} = \bigg (2s_{2}\mathcal {R}^{-1}U-s_{1}\bigg )\bigg (2\mathcal {R}-s_{1}U\bigg )^{-1},$ is holomorphic on $r\cdot \mathbb {G}$. Thus statement (1) is proved.

To prove the second statement, note that

$$ s_{U,\mathcal {R}} = \bigg (s_{2}\mathcal {R}^{-1}U-\dfrac{1}{2}s_{1}\bigg )\bigg (1_{\mathcal {M}}-\dfrac{s_{1}}{2}\mathcal {R}^{-1}U\bigg )^{-1}\mathcal {R}^{-1}. $$

Since $s=(s_{1},s_{2})\in r\cdot \mathbb {G}$, there is $q = (q_{1},q_{2}) \in \mathbb {G}$ such that $s_{1}=rq_1$ and $s_2 =r^2 q_2$. Thus, for $s=(s_{1},s_{2})\in r\cdot \mathbb {G}$,

$$\begin{aligned} s_{U,\mathcal {R}}&= \bigg ( r^{2}q_{2} \begin{bmatrix} 1_{\mathcal {H}_{1}} &  0 \\ 0 &  r^{-1}_{\mathcal {H}_{1}^{\perp }} \\ \end{bmatrix}U-\dfrac{1}{2}q_{1}r\bigg )\bigg (1_{\mathcal {M}}- \begin{bmatrix} 1_{\mathcal {H}_{1}} &  0 \\ 0 &  r^{-1}_{\mathcal {H}_{1}^{\perp }} \\ \end{bmatrix}\dfrac{1}{2}q_{1}rU\bigg )^{-1}\mathcal {R}^{-1} \\&=r\bigg ( rq_{2}\begin{bmatrix} 1_{\mathcal {H}_{1}} &  0 \\ 0 &  r^{-1}_{\mathcal {H}_{1}^{\perp }} \\ \end{bmatrix}U-\dfrac{1}{2}q_{1}\bigg )\bigg (1_{\mathcal {M}}- \begin{bmatrix} 1_{\mathcal {H}_{1}} &  0 \\ 0 &  r^{-1}_{\mathcal {H}_{1}^{\perp }} \\ \end{bmatrix}\dfrac{1}{2}q_{1}rU\bigg )^{-1}\mathcal {R}^{-1} \\&=\bigg ( q_{2}\begin{bmatrix} r_{\mathcal {H}_{1}} &  0 \\ 0 &  1_{\mathcal {H}_{1}^{\perp }} \\ \end{bmatrix}U-\dfrac{1}{2}q_{1}\bigg )\bigg (1_{\mathcal {M}}- \begin{bmatrix} r_{\mathcal {H}_{1}} &  0 \\ 0 &  1_{\mathcal {H}^{\perp }} \\ \end{bmatrix}\dfrac{1}{2}q_{1}U\bigg )^{-1}\bigg ( r\mathcal {R}^{-1}\bigg ) \\&= \bigg ( q_{2} \begin{bmatrix} r_{\mathcal {H}_{1}} &  0 \\ 0 &  1_{\mathcal {H}_{1}^{\perp }} \\ \end{bmatrix}U-\dfrac{1}{2}q_{1}\bigg )\bigg (1_{\mathcal {M}}- \begin{bmatrix} r_{\mathcal {H}_{1}} &  0 \\ 0 &  1_{\mathcal {H}_{1}^{\perp }} \\ \end{bmatrix}\dfrac{1}{2}q_{1}U\bigg )^{-1}\begin{bmatrix} r_{\mathcal {H}_{1}} &  0 \\ 0 &  1_{\mathcal {H}_{1}^{\perp }} \\ \end{bmatrix}. \end{aligned}$$

For all $q=(q_{1},q_{2})\in \mathbb {G}$, define

$$f_{q}(\lambda ) = \dfrac{q_{2}\lambda -\dfrac{1}{2}q_{1}}{1-\dfrac{1}{2}q_{1}\lambda },$$

for $\lambda$ in a neighbourhood of $\overline{\mathbb {D}}$. The linear fractional map $f_{q}$ maps $\mathbb {D}$ onto the open disc with centre and radius

$$ 2\dfrac{\overline{q}_{1}q_{2} -q_{1}}{4-|{q_{1}}|^{2}}, ~~\dfrac{|{q_{1}^{2}-4q_{2}|}}{4-|{q_{1}|}^{2}},$$

respectively.

Note that the operator

$$\begin{bmatrix} r\cdot 1_{\mathcal {H}_{1}} &  0 \\ 0 &  1_{\mathcal {H}^{\perp }_{1}} \\ \end{bmatrix}U$$

is a contraction on $\mathcal {M}$ and

$$ s_{U,\mathcal {R}} = f_{q}\bigg ( \begin{bmatrix} r\cdot 1_{\mathcal {H}_{1}} &  0 \\ 0 &  1_{\mathcal {H}^{\perp }_{1}} \\ \end{bmatrix}U\Bigg ) \begin{bmatrix} r\cdot 1_{\mathcal {H}_{1}} &  0 \\ 0 &  1_{\mathcal {H}^{\perp }_{1}} \\ \end{bmatrix}. $$

By von Neumann’s inequality, we have

2.35 $$\begin{aligned} \Vert s_{U,\mathcal {R}} \Vert _{\mathcal {B(M)}}= &   \Bigg \Vert f_{q}\bigg ( \begin{bmatrix} r\cdot 1_{\mathcal {H}_{1}} &  0 \\ 0 &  1_{\mathcal {H}^{\perp }_{1}} \\ \end{bmatrix}U\Bigg ) \begin{bmatrix} r\cdot 1_{\mathcal {H}_{1}} &  0 \\ 0 &  1_{\mathcal {H}^{\perp }_{1}} \\ \end{bmatrix}\Bigg \Vert \nonumber \\\le &   \Bigg \Vert f_{q}\bigg ( \begin{bmatrix} r\cdot 1_{\mathcal {H}_{1}} &  0 \\ 0 &  1_{\mathcal {H}^{\perp }_{1}} \\ \end{bmatrix}U\Bigg ) \Bigg \Vert \nonumber \\\le &   \sup _{\mathbb {D}} |{f_{q}|} = \dfrac{ 2|{\overline{q}_{1}q_{2} -q_{1}|}+|{q_{1}^{2}-4q_{2}|}}{4-|{q_{1}|}^{2}}. \end{aligned}$$

By [2, Theorem 2.1], the right hand side of inequality (2.35) is less than one for all $q\in \mathbb {G}$. Thus statement (2) is proved.

(3). In part (1), we have shown that

$$w:r\cdot \mathbb {G}\rightarrow \mathcal {B}(\mathcal {M}): \ s \mapsto s_{U,\mathcal {R}}$$

is holomorphic on $r\cdot \mathbb {G}$. Hence, for every contraction $D \in {\mathcal {B(M)}}$, the map $s \mapsto 1_{\mathcal {M}}-Ds_{U,\mathcal {R}}$ is holomorphic on $r\cdot \mathbb {G}$. By part (2), for every $s \in r\cdot \mathbb {G}$, $\Vert s_{U,\mathcal {R}}\Vert _{\mathcal {B(M)}}< 1$. Thus $1_{\mathcal {M}}-Ds_{U,\mathcal {R}}$ is invertible. Therefore, by [6, Proposition I.2.6], for every $\gamma \in \mathcal {M}$, the $\mathcal {M}$-valued function

$$u:r\cdot \mathbb {G}\rightarrow \mathcal {M}, \ \text { defined by} u(s) = (1_{\mathcal {M}}-Ds_{U,\mathcal {R}})^{-1}\gamma ,$$

is holomorphic on $r\cdot \mathbb {G}$. $\square$

## A Model Formula and a Realization for the Symmetrized Skew Bidisc

Let us use Theorem 2.11 to show that there is a model formula for a function in $\mathcal {S}(\mathbb {G}_{r})$.

### Theorem 3.1

Let $r\in (0,1)$ and let $f\in \mathcal {S}(\mathbb {G}_{r})$. Then there exist a model $(\mathcal {M}, (U,\mathcal {R}), u)$ for *f* on $r\cdot \mathbb {G}$, that is, there exist a complex Hilbert space $\mathcal {M}$, a closed non-trivial proper subspace $\mathcal {H}_{1}$ of $\mathcal {M}$, a holomorphic map $u:r\cdot \mathbb {G}\rightarrow \mathcal {M}$, a unitary operator *U* on $\mathcal {M}$ and the operator $\mathcal {R}$ on $\mathcal {M}$ defined by

3.2 $$\begin{aligned} \mathcal {R}=\begin{bmatrix} 1_{\mathcal {H}_{1} } &  0 \\ 0 &  r\cdot 1_{\mathcal {H}_{1}^{\perp }} \\ \end{bmatrix}, \end{aligned}$$

such that, for all $s=(s_{1},s_{2})\in r\cdot \mathbb {G}$ and $t=(t_{1},t_{2})\in r \cdot \mathbb {G}$,

3.3 $$\begin{aligned} 1- \overline{f(t)}f(s) =\Bigg \langle \bigg (1_{\mathcal {M}}-t_{U,\mathcal {R}}^{*}s_{U,\mathcal {R}}\bigg )u(s),u(t)\Bigg \rangle _{\mathcal {M}}, \end{aligned}$$

where the operators $s_{U,\mathcal {R}}$ and $t_{U,\mathcal {R}}$ are strict contractions on $\mathcal {M}$ defined by equation (2.34).

### Remark 3.4

Note that in this theorem we only prove that the formula (3.3) is valid on $r \cdot \mathbb {G}$, which is a proper subset of $\mathbb {G}_{r}$, since we can only guarantee that $s_{U,\mathcal {R}}$ given by equation (2.34) and *u* are well defined on $r \cdot \mathbb {G}$.

### Proof

For the given $f\in \mathcal {S}(\mathbb {G}_{r})$, we define $F=f\circ \pi \circ T_{r} : \mathbb {D}^{2} \rightarrow \overline{\mathbb {D}}$, see equations (2.7), (2.6) and (2.9). By Theorem 2.11, there exists a Hilbert space $\mathcal {M} = \mathcal {H}_{1}\oplus \mathcal {H}_{2}$, a unitary operator *U* on $\mathcal {M}$, and a holomorphic map $w: r\mathbb {D}\times \mathbb {D} \rightarrow \mathcal {M}$, which satisfies $w(\lambda ^{\sigma }) = w(\lambda )$ for all $\lambda \in r\mathbb {D}\times \mathbb {D}$, such that, for all $\lambda , \mu \in r\mathbb {D}\times \mathbb {D}$,

3.5 $$\begin{aligned} 1-\overline{F(\mu )}F(\lambda ) = \langle Z_{r}(\lambda , \mu ) w(\lambda ),w(\mu )\rangle _{\mathcal {M}}, \end{aligned}$$

where

3.6 $$\begin{aligned} Z_{r}(\lambda ,\mu )= &   (1_{\mathcal {M}}-r\overline{\mu _{2}}\mathcal {R}^{-1}U^{*})(1_{\mathcal {M}}-\overline{\mu }_{1} \lambda _{1}\mathcal {R}^{-2})(1_{\mathcal {M}}-r\lambda _{2}U\mathcal {R}^{-1})\nonumber \\  &   + (1_{\mathcal {M}}-\overline{\mu _{1}}\mathcal {R}^{-1}U^{*})(1_{\mathcal {M}}-r^{2} \overline{\mu }_{2}\lambda _{2}\mathcal {R}^{-2})(1_{\mathcal {M}}-\lambda _{1}U\mathcal {R}^{-1}). \end{aligned}$$

Let us rewrite $Z_{r}$ with symmetric variables with respect to $\sigma$ in $r \cdot \mathbb {G}$. For $\lambda , \mu \in r\mathbb {D}\times \mathbb {D}$, expand equation (3.6),

$$\begin{aligned}&Z_{r}(\lambda ,\mu ) \\&\quad = (1_{\mathcal {M}}-\overline{\mu _{1}}\lambda _{1}\mathcal {R}^{-2}-r\overline{\mu _{2}}\mathcal {R}^{-1}U^{*} + r\overline{\mu _{1}}\overline{\mu _{2}}\lambda _{1}\mathcal {R}^{-1}U^{*}\mathcal {R}^{-2})(1_{\mathcal {M}}-r\lambda _{2}U\mathcal {R}^{-1})\\&\qquad +(1_{\mathcal {M}}-r^{2}\overline{\mu _{2}}\lambda _{2}\mathcal {R}^{-2}-\overline{\mu _{1}}\mathcal {R}^{-1}U^{*}+r^{2}\overline{\mu _{1}}\overline{\mu _{2}}\lambda _{2}\mathcal {R}^{-1}U^{*}\mathcal {R}^{-2})(1_{\mathcal {M}}-\lambda _{1}U\mathcal {R}^{-1})\\&\quad = 1_{\mathcal {M}}-r\lambda _{2}U\mathcal {R}^{-1}-\overline{\mu _{1}}\lambda _{1}\mathcal {R}^{-2}+r\overline{\mu _{1}}\lambda _{1}\lambda _{2}\mathcal {R}^{-2}U\mathcal {R}^{-1}-r\overline{\mu _{2}}\mathcal {R}^{-1}U^{*}\\&\qquad +r^{2}\overline{\mu _{2}}\lambda _{2}R^{-1}U^{*}UR^{-1}+r\overline{\mu _{1}}\overline{\mu _{2}}\lambda _{1}\mathcal {R}^{-1}U^{*}\mathcal {R}^{-2}-r^{2}\overline{\mu _{1}}\overline{\mu _{2}}\lambda _{1}\lambda _{2}\mathcal {R}^{-1}U^{*}\mathcal {R}^{-2}U\mathcal {R}^{-1}\\&\qquad +1_{\mathcal {M}}-\lambda _{1}U\mathcal {R}^{-1}-r^{2}\overline{\mu _{2}}\lambda _{2}\mathcal {R}^{-2}+r^{2}\overline{\mu _{2}}\lambda _{1}\lambda _{2}\mathcal {R}^{-2}U\mathcal {R}^{-1}-\overline{\mu _{1}}\mathcal {R}^{-1}U^{*}\\&\qquad +\overline{\mu _{1}}\lambda _{1}\mathcal {R}^{-1}U^{*}U\mathcal {R}^{-1}+r^{2}\overline{\mu _{1}}\overline{\mu _{2}}\lambda _{2}\mathcal {R}^{-1} U^{*}\mathcal {R}^{-2}-r^{2}\overline{\mu _{1}}\overline{\mu _{2}}\lambda _{1}\lambda _{2}\mathcal {R}^{-1}U^{*}\mathcal {R}^{-2} U \mathcal {R}^{-1}. \end{aligned}$$

Since *U* is unitary, let us simplify and collect terms to find that

3.7 $$\begin{aligned} Z_{r}(\lambda ,\mu )= &   2\Big (1_{\mathcal {M}}-r^{2}\overline{\mu }_{1}\overline{\mu }_{2}\lambda _{1}\lambda _{2}\mathcal {R}^{-1}U^{*}\mathcal {R}^{-2}U\mathcal {R}^{-1}\Big ) \nonumber \\  &   +\Big ( r\lambda _{1}\lambda _{2}(\overline{\mu _{1}}+r\overline{\mu _{2}})\mathcal {R}^{-2}-(\lambda _{1}+r\lambda _{2})\Big )U\mathcal {R}^{-1} \nonumber \\  &   +\mathcal {R}^{-1}U^{*}\Big (r\overline{\mu }_{1} \overline{\mu }_{2}(\lambda _{1}+r\lambda _{2})\mathcal {R}^{-2}-(\overline{\mu _{1}}+r\overline{\mu _{2}})\Big ), \end{aligned}$$

for $$\lambda ,\mu \in r\mathbb {D}\times \mathbb {D}$$.

Thus, for $\lambda , \mu \in r\mathbb {D}\times \mathbb {D}$, we introduce symmetric variables with respect to $\sigma$

3.8 $$\begin{aligned}  &   s_{1}=\lambda _{1}+r\lambda _{2},~s_{2} = r\lambda _{1}\lambda _{2} \nonumber \\  &   t_{1}=\mu _{1}+r\mu _{2},~t_{2}=r\mu _{1}\mu _{2}. \end{aligned}$$

It is clear that $s =(s_1, s_2)$ and $t=(t_1,t_2)$ are in $r \cdot \mathbb {G}$ and,

3.9 $$\begin{aligned} (s^{\sigma })^{\sigma } = (r s_{2},r^{-1} s_{1})^{\sigma } = (rr^{-1} s_{1},r^{-1} r s_{2}) = s, \end{aligned}$$

3.10 $$\begin{aligned} (t^{\sigma })^{\sigma } = (r t_{2},r^{-1} t_{1})^{\sigma } = (rr^{-1} t_{1},r^{-1} r t_{2}) = t. \end{aligned}$$

We can rewrite equation (3.7) in terms of $(s_{1},s_{2}), (t_{1},t_{2}) \in r \cdot \mathbb {G}$ using connections (3.8), to obtain

3.11 $$\begin{aligned} Z_{r}(\lambda ,\mu )= &   Y_{\mathcal {R},U}(s,t) = 2\Big (1_{\mathcal {M}}-\overline{t}_{2}s_{2}\mathcal {R}^{-1}U^{*}\mathcal {R}^{-2}U\mathcal {R}^{-1}\Big )\nonumber \\  &   +\Big (\overline{t}_{1}s_{2}\mathcal {R}^{-2}-s_{1}\Big )U\mathcal {R}^{-1} +\mathcal {R}^{-1}U^{*}\Big (\overline{t}_{2}s_{1}\mathcal {R}^{-2}-\overline{t}_{1}\Big ). \end{aligned}$$

One can check that

$$\begin{aligned} Y_{\mathcal {R},U}(s,t) =&\dfrac{1}{2}\bigg (2-\overline{t}_{1}\mathcal {R}^{-1}U^{*}\bigg )\bigg (2-s_{1}U\mathcal {R}^{-1}\bigg )\\&-\dfrac{1}{2}\mathcal {R}^{-1}\bigg (2\overline{t}_{2}U^{*}\mathcal {R}^{-1}-\overline{t}_{1}\bigg )\bigg (2s_{2}\mathcal {R}^{-1}U-s_{1}\bigg )\mathcal {R}^{-1}. \end{aligned}$$

Recall Definition 2.34 of the operator $s_{U,\mathcal {R}}$ on $\mathcal {M}$:

$$\begin{aligned} s_{U,\mathcal {R}} = \bigg (2s_{2}\mathcal {R}^{-1}U-s_{1}\bigg )\bigg (2\mathcal {R}-s_{1}U\bigg )^{-1} \end{aligned}$$

for $s=(s_{1},s_{2}) \in r \cdot \mathbb {G}$. By Lemma 2.32, the operator $s_{U,\mathcal {R}}$ is well defined and is a strict contraction for all $s\in r\cdot \mathbb {G}$. We can check that, for $s,t \in r\cdot \mathbb {G}$,

3.12 $$\begin{aligned} Y_{\mathcal {R},U}(s,t) = \dfrac{1}{2}\bigg (2-t_{1}U\mathcal {R}^{-1}\bigg )^{*}\bigg (1_{\mathcal {M}}-t_{U,\mathcal {R}}^{*}s_{U,\mathcal {R}}\bigg )\bigg (2-s_{1}U\mathcal {R}^{-1}\bigg ). \end{aligned}$$

Moreover, note that *w* in equation (3.5) respects the symmetry of the involution $\sigma$ by equation (2.31). Hence there exists a holomorphic function $x: r\cdot \mathbb {G}\rightarrow \mathcal {M}$ such that, for all $\lambda \in r\mathbb {D}\times \mathbb {D}$,

$$ w(\lambda ) = x(\lambda _{1}+r\lambda _{2}, r\lambda _{1}\lambda _{2}) = x(s_{1},s_{2})=x(s),$$

using the relations (3.8). Recall that for $f\in \mathcal {S}(\mathbb {G}_{r})$, we have defined

$$F=f\circ \pi \circ T_{r} : \mathbb {D}^{2} \rightarrow \overline{\mathbb {D}},$$

and so, for $\lambda \in r\mathbb {D}\times \mathbb {D}$,

3.13 $$\begin{aligned} F(\lambda )= f(\lambda _{1}+r\lambda _{2},r\lambda _{1}\lambda _{2})= f(s_1, s_2)= f(s), \end{aligned}$$

where *s* is defined by equations (3.8). Therefore, using equations (3.13) and (3.11), we can re-write the equation (3.5) in the following form

$$ 1-\overline{f(t)}f(s) = \bigg \langle Y_{\mathcal {R},U}(s,t) x(s), x(t)\bigg \rangle _{\mathcal {M}},$$

for all $s,t\in r\cdot \mathbb {G}$. Hence, by equation (3.12),

$$1-\overline{f(t)}f(s) = \Bigg \langle \dfrac{1}{2} \bigg (2-t_{1}U\mathcal {R}^{-1}\bigg )^{*}\bigg (1_{\mathcal {M}}-t_{U,\mathcal {R}}^{*}s_{U,\mathcal {R}}\bigg )\bigg (2-s_{1}U\mathcal {R}^{-1}\bigg )x(s),x(t)\Bigg \rangle _{\mathcal {M}}$$

and

3.14 $$\begin{aligned} 1  &   - \overline{f(t)}f(s)= \nonumber \\  &   \Bigg \langle \bigg (1_{\mathcal {M}}-t_{U,\mathcal {R}}^{*}s_{U,\mathcal {R}}\bigg )\dfrac{1}{\sqrt{2}}\bigg (2-s_{1}U\mathcal {R}^{-1}\bigg )x(s),\dfrac{1}{\sqrt{2}}\bigg (2-t_{1}U\mathcal {R}^{-1}\bigg )x(t)\Bigg \rangle _{\mathcal {M}}, \end{aligned}$$

for all $s,t\in r\cdot \mathbb {G}$. Define a holomorphic map $u:r\cdot \mathbb {G}\rightarrow \mathcal {M}$, by

3.15 $$\begin{aligned} u(s) = \dfrac{1}{\sqrt{2}}\bigg (2-s_{1}U\mathcal {R}^{-1}\bigg )x(s), \ \text {for all} \ s \in r\cdot \mathbb {G}. \end{aligned}$$

Thus, by equation (3.14),

$$1-\overline{f(t)}f(s) = \Bigg \langle \bigg (1_{\mathcal {M}}-t_{U,\mathcal {R}}^{*}s_{U,\mathcal {R}}\bigg )u(s),u(t)\Bigg \rangle _{\mathcal {M}} \ \text {for all} \ s, t \in r\cdot \mathbb {G}.$$

Therefore equation (3.3) is proved.$\square$

Theorem 3.1 allows us to find a realization for functions in $\mathcal {S}(\mathbb {G}_{r})$.

### Theorem 3.16

Let $r\in (0,1)$ and $f \in \mathcal {S}(\mathbb {G}_{r})$. There exist a scalar *a*, a complex Hilbert space $\mathcal {M}$, a closed non-trivial proper subspace $\mathcal {H}_{1}$ of $\mathcal {M}$, vectors $\beta , \gamma , \in \mathcal {M}$, operators *D* and *U* on $\mathcal {M}$ such that *U* is unitary and the operator

3.17 $$\begin{aligned} L=\begin{bmatrix} a &  1\otimes \beta \\ \gamma \otimes 1 &  D \end{bmatrix} \end{aligned}$$

is unitary on $\mathbb {C}\oplus \mathcal {M}$ and, for all $s=(s_{1},s_{2})\in r\cdot \mathbb {G}$,

3.18 $$\begin{aligned} f(s)=a+\langle s_{U,\mathcal {R}}(1_{\mathcal {M}}-Ds_{U,\mathcal {R}})^{-1}\gamma , \beta \rangle _{\mathcal {M}}, \end{aligned}$$

where the operator $s_{U,\mathcal {R}}$ is defined by equation (2.34) and the operator $\mathcal {R} \in \mathcal {B(M)}$ given by equation (3.2).

### Proof

By Theorem 3.1, there exists a Hilbert space $\mathcal {M}=\mathcal {H}_{1}\oplus \mathcal {H}_{2}$, a holomorphic map $u: r\cdot \mathbb {G} \rightarrow \mathcal {M}$, a unitary operator *U* on $\mathcal {M}$ and an operator $\mathcal {R}\in \mathcal {B}(\mathcal {M})$ given by equation (3.2), such that, for all $s,t \in r\cdot \mathbb {G}$,

3.19 $$\begin{aligned} 1- \overline{f(t)}f(s) =\Bigg \langle \bigg (1_{\mathcal {M}}-t_{U,\mathcal {R}}^{*}s_{U,\mathcal {R}}\bigg )u(s),u(t)\Bigg \rangle _{\mathcal {M}}. \end{aligned}$$

Rearrange equation (3.19) to show that, for all $s,t \in r\cdot \mathbb {G}$,

$$ 1+ \langle s_{U,\mathcal {R}} u(s),t_{U,\mathcal {R}}u(t)\rangle _{\mathcal {M}} = \langle f(s), f(t) \rangle _{\mathbb {C}}+ \langle u(s), u(t) \rangle _{\mathcal {M}}, $$

which is equivalent to

3.20 $$\begin{aligned} \Bigg \langle \begin{bmatrix} 1 \\ s_{U,\mathcal {R}}u(s)\\ \end{bmatrix}, \begin{bmatrix} 1 \\ t_{U,\mathcal {R}}u(t)\\ \end{bmatrix} \Bigg \rangle _{\mathbb {C}\oplus \mathcal {M}} = \Bigg \langle \begin{bmatrix} f(s) \\ u(s)\\ \end{bmatrix}, \begin{bmatrix} f(t) \\ u(t)\\ \end{bmatrix} \Bigg \rangle _{\mathbb {C}\oplus \mathcal {M}}. \end{aligned}$$

This means that the two families of vectors

$$\begin{aligned} \begin{bmatrix} 1 \\ s_{U,\mathcal {R}}u(s) \end{bmatrix}_{s\in r\cdot \mathbb {G}}~\text {and}~ \begin{bmatrix} f(s) \\ u(s) \end{bmatrix}_{s\in r\cdot \mathbb {G}} \end{aligned}$$

have the same Gramians in $\mathbb {C}\oplus \mathcal {M}$. Hence there exists a linear isometry $L\in \mathcal {B}(\mathbb {C}\oplus \mathcal {M})$ such that

$$\begin{aligned} L : \overline{{{\,\textrm{Span}\,}}}\Bigg \{ \begin{bmatrix} 1 \\ s_{U,\mathcal {R}}u(s)\\ \end{bmatrix} : s \in r\cdot \mathbb {G} \Bigg \}\rightarrow \overline{{{\,\textrm{Span}\,}}}\Bigg \{ \begin{bmatrix} f(s) \\ u(s)\\ \end{bmatrix}: s \in r\cdot \mathbb {G} \Bigg \}, \end{aligned}$$

and

3.21 $$\begin{aligned} L\begin{bmatrix} 1 \\ s_{U,\mathcal {R}}u(s)\\ \end{bmatrix} = \begin{bmatrix} f(s) \\ u(s)\\ \end{bmatrix}, \end{aligned}$$

for all $s\in r\cdot \mathbb {G}$. Enlarge the Hilbert space $\mathcal {M}$ if necessary, and simultaneously the unitary operator *U* and the operator $\mathcal {R}$ on $\mathcal {M}$, so that the isometry *L* extends to a unitary operator

3.22 $$\begin{aligned} \tilde{L} = \begin{bmatrix} a &  1 \otimes \beta \\ \gamma \otimes 1 &  D \end{bmatrix}, \end{aligned}$$

on $\mathbb {C}\oplus \mathcal {M}$ for some vectors $\beta , \gamma \in \mathcal {M}$, $a\in \mathbb {C}$ and a contraction $D \in \mathcal {B(M)}$. By equation (3.21), for every $s\in r\cdot \mathbb {G}$,

$$\begin{aligned} f(s)&= a+(1\otimes \beta ) s_{U,\mathcal {R}}u(s), \\ u(s)&= (\gamma \otimes 1)(1) + D s_{U,\mathcal {R}}u(s). \end{aligned}$$

Thus, for every $s\in r\cdot \mathbb {G}$,

3.23 $$\begin{aligned} f(s)= &   a+\langle s_{U,\mathcal {R}}u(s), \beta \rangle _{\mathcal {M}}, \nonumber \\ u(s)= &   \gamma +Ds_{U,\mathcal {R}}u(s). \end{aligned}$$

Since *D* is a contraction and by Lemma 2.32, $\Vert s_{U,\mathcal {R}}\Vert _{\mathcal {B(M)}}< 1$ for all $s\in r\cdot \mathbb {G}$, we deduce that the operator $(1_{\mathcal {M}}-Ds_{U,\mathcal {R}})$ is invertible for all $s\in r\cdot \mathbb {G}$. Therefore

$$ u(s) = (1_{\mathcal {M}}-Ds_{U,\mathcal {R}})^{-1}\gamma , \ \text {for} \ s\in r\cdot \mathbb {G},$$

and so we can eliminate *u*(*s*) from the system of equations (3.23) to get the following formula

$$f(s)=a+\langle s_{U,\mathcal {R}}(1_{\mathcal {M}}-Ds_{U,\mathcal {R}})^{-1}\gamma , \beta \rangle _{\mathcal {M}},$$

for all $$s\in r\cdot \mathbb {G}$$. $$\square $$

We now show that every function $f:r\cdot \mathbb {G} \rightarrow \mathbb {C}$ that has a realization formula (3.18) belongs to $\mathcal {S}(r\cdot \mathbb {G})$.

### Theorem 3.24

Let $\mathcal {M}$ be a complex Hilbert space, let $\mathcal {H}_{1}$ be a closed non-trivial proper subspace, let $\beta , \gamma \in \mathcal {M}$ and let *D* and *U* be operators on $\mathcal {M}$ such that *U* is unitary, the operator

3.25 $$\begin{aligned} L=\begin{bmatrix} a &  1\otimes \beta \\ \gamma \otimes 1 &  D \end{bmatrix} \end{aligned}$$

is unitary on $\mathbb {C}\oplus \mathcal {M}$ and let $f:r\cdot \mathbb {G}\rightarrow \mathbb {C}$ be defined by

3.26 $$\begin{aligned} f(s)=a+\langle s_{U,\mathcal {R}}(1_{\mathcal {M}}-Ds_{U,\mathcal {R}})^{-1}\gamma , \beta \rangle _{\mathcal {M}}~~\text {for all}~s\in r\cdot \mathbb {G}, \end{aligned}$$

where

3.27 $$\begin{aligned} s_{U,\mathcal {R}} = \bigg (2s_{2}\mathcal {R}^{-1}U-s_{1}\bigg )\bigg (2\mathcal {R}-s_{1}U\bigg )^{-1} \end{aligned}$$

and the operator $\mathcal {R}\in \mathcal {B}(\mathcal {M})$ is given by equation (3.2). Then $f\in \mathcal {S}(r\cdot \mathbb {G})$.

### Proof

Let us show that the map *f* given by equation (3.26) is well defined and holomorphic on $r\cdot \mathbb {G}$. By Lemma 2.32 (1) and (2), the operator-valued function

$$w:r\cdot \mathbb {G}\rightarrow \mathcal {B(M)} : s\mapsto s_{U,\mathcal {R}},$$

is well defined and holomorphic on $r\cdot \mathbb {G}$ and $\Vert s_{U,\mathcal {R}}\Vert _{\mathcal {B(M)}}< 1$ for all $s=(s_{1},s_{2})\in r\cdot \mathbb {G}$. Since *L* is a unitary matrix, $\Vert D\Vert _{\mathcal {B}(\mathcal {M})}\le 1.$ Therefore, by Lemma 2.32 (3), for every $\gamma \in \mathcal {M}$, the $\mathcal {M}$-valued function

$$u:r\cdot \mathbb {G}\rightarrow \mathcal {M} \ \text { defined by} \ u(s) = (1_{\mathcal {M}}-Ds_{U,\mathcal {R}})^{-1}\gamma $$

is holomorphic on $r\cdot \mathbb {G}$. Hence, *f* is holomorphic on $r\cdot \mathbb {G}$.

To prove that $|f(s)|\le 1$ on $r \cdot \mathbb {G}$, note that for all $s\in r\cdot \mathbb {G}$,

$$ L\begin{bmatrix} 1 \\ s_{U,\mathcal {R}}u(s) \end{bmatrix} = \begin{bmatrix} a+(1\otimes \beta )s_{U,\mathcal {R}} \\ \gamma +Ds_{U,\mathcal {R}}u(s) \end{bmatrix}= \begin{bmatrix} f(s) \\ u(s) \end{bmatrix}.$$

Since *L* is unitary,

$$\bigg \langle \begin{bmatrix} f(s)\\ u(s) \end{bmatrix}, \begin{bmatrix} f(t)\\ u(t) \end{bmatrix} \bigg \rangle _{\mathbb {C}\oplus \mathcal {M}} = \bigg \langle \begin{bmatrix} 1\\ s_{U,\mathcal {R}}u(s) \end{bmatrix}, \begin{bmatrix} 1\\ t_{U,\mathcal {R}}u(t) \end{bmatrix} \bigg \rangle _{\mathbb {C}\oplus \mathcal {M}}~\text {for all}~s,t\in r\cdot \mathbb {G}. $$

By a reshuffle of the above equation, this defines a model $(\mathcal {M},u)$ for the function *f* on $r\cdot \mathbb {G}$, that is,

$$1-\overline{f(t)}f(s) = \bigg \langle (1_{\mathcal {M}}-t^{*}_{U,\mathcal {R}}s_{U,\mathcal {R}})u(s),u(t)\bigg \rangle _{\mathcal {M}}~~\text {for}~s,t\in r\cdot \mathbb {G}.$$

Let $t=s$ in the model equation above for *f*. Then

$$1-|f(s) |^{2} =\bigg \langle (1_{\mathcal {M}}-s^{*}_{U,\mathcal {R}}s_{U,\mathcal {R}})u(s),u(s)\bigg \rangle _{\mathcal {M}}.$$

Since $s_{U,\mathcal {R}}$ is a strict contraction for all $s\in r\cdot \mathbb {G}$, we have $1-s^{*}_{U,\mathcal {R}}s_{U,\mathcal {R}}\ge 0$ and thus

$$1-|f(s) |^{2} \ge 0~\text {for all}~~s\in r\cdot \mathbb {G}.$$

Hence $$f\in \mathcal {S}(r\cdot \mathbb {G})$$.$$\square $$

## A Realization Formula and a Pick Theorem for Functions in $\mathcal {S}(r\cdot \mathbb {G})$

When we restrict our attention to functions from $\mathcal {S}(r\cdot \mathbb {G})$, we can use a different method to derive a realization formula and a Pick theorem for them.

Let $r\in (0,1)$. There exists a biholomorphic “scaling map” between $\mathbb {G}$ and $r\cdot \mathbb {G}$

$$ \psi _r: \mathbb {G} \rightarrow r\cdot \mathbb {G} \ \text {given by} \ \psi _r(z_1, z_2)= (rz_1, r^2 z_2).$$

Hence we can deduce a number of statements about $f\in \mathcal {S}(r \cdot \mathbb {G})$ directly from known facts about holomorphic functions on $\mathbb {G}$, see [5].

For example, if $f\in \mathcal {S}(r \cdot \mathbb {G})$, then $f\circ \psi _r \in \mathcal {S}(\mathbb {G})$, and so, by [5, Theorem 2.2], $f\circ \psi _r$ has a $\mathbb {G}$-model $(\mathcal {M},T,u)$, where $\mathcal {M}$ is a Hilbert space, *T* is a contraction acting on $\mathcal {M}$ and $u:\mathbb {G} \rightarrow \mathcal {M}$ is a holomorphic function such that, for all $q,p\in \mathbb {G}$,

4.1 $$\begin{aligned} 1-\overline{f\circ \psi _r (p)}f\circ \psi _r (q)= \langle (1-p_T^* q_T) u(q),u(p)\rangle _\mathcal {M}. \end{aligned}$$

Here, for any point $q=(q_1,q_2)\in \mathbb {G}$ and any contractive linear operator *T* on a Hilbert space $\mathcal {M}$, the operator $q_T$ is defined by

4.2 $$\begin{aligned} q_T=(2q_2T-q_1)(2-q_1 T)^{-1}\quad \text{ on } \mathcal {M}. \end{aligned}$$

For any $s,t\in r \cdot \mathbb {G}$, apply formula (4.1) to $q= \psi _r^{-1} (s), p = \psi _r^{-1} (t)$ and observe that

4.3 $$\begin{aligned} q_T= (\psi _r^{-1} (s))_T=(2 r^{-2} s_2 T-r^{-1}s_1)(2-r^{-1}s_1 T)^{-1}= r^{-1} s_{r^{-1} T} \quad \text{ on } \mathcal {M}. \end{aligned}$$

Note that the operator $s_{r^{-1} T}$ is well defined for $s\in r \cdot \mathbb {G}$ and a contractive linear operator *T*. Then equation (4.1) implies that, for all $s,t\in r \cdot \mathbb {G}$,

4.4 $$\begin{aligned} 1-\overline{f(s)}f(t)= \langle (1- r^{-2} t_{r^{-1} T}^* s_{r^{-1} T}) u(\psi _r^{-1} (s)),u(\psi _r^{-1} (t))\rangle _\mathcal {M}. \end{aligned}$$

Therefore we obtain a model formula $$(\mathcal {M},X,v)$$ for $$f\in \mathcal {S}(r \cdot \mathbb {G})$$, where $$\mathcal {M}$$ is a Hilbert space, $$X=r^{-1} T $$ is an operator acting on $$\mathcal {M}$$ with $$\Vert X\Vert \le r^{-1}$$ and $$v:r \cdot \mathbb {G} \rightarrow \mathcal {M}$$, given by $$v = u \circ \psi _r^{-1}$$, is a holomorphic function such that, for all $$s,t\in r \cdot \mathbb {G}$$,

4.5 $$\begin{aligned} 1-\overline{f(s)}f(t)= \langle (1- r^{-2} t_{X}^* s_{X}) v(s),v (t)\rangle _\mathcal {M}. \end{aligned}$$

We can also use known facts about functions from $\mathcal {S}(\mathbb {G})$ get a realization formula for functions from $\mathcal {S}(r \cdot \mathbb {G})$ and a natural variant of the classical Pick interpolation theorem in which the interpolation nodes lie in $r \cdot \mathbb {G}$.

### Theorem 4.6

Let $r\in (0,1)$. Let $s_{1},s_{2},\ldots s_{n}$ be distinct points in $r\cdot \mathbb {G}$ and let $w_{1},w_{2},\ldots ,w_{n}$ be points in $\overline{\mathbb {D}}$. The following statements are equivalent:

1. There exists a holomorphic function $\varphi \in \mathcal {S}(r\cdot \mathbb {G})$ such that $\varphi (s_{i})=w_{i}$ for $i=1,\ldots ,n$;
2. There exist a Hilbert space $\mathcal {M}$, an operator *X* on $\mathcal {M}$ with $\Vert X\Vert \le r^{-1}$ and vectors $v_{1},v_{2},\ldots ,v_{n}$ in $\mathcal {M}$ such that
  4.7 $$\begin{aligned} 1-\overline{w_{i}}w_{j}=\bigg \langle \Big (1_{\mathcal {M}}-r^{-2} (s_{i})^{*}_{X} (s_{j})_{X}\Big )v_{j},v_{i}\bigg \rangle _{\mathcal {M}} \end{aligned}$$
  for $$i,j=1,\ldots ,n$$.

### Proof

It follows from Theorem 5.1 of [5]. $\square$

## Examples of Functions in $\mathcal {S}(r\cdot \mathbb {G})$

We now make use of the realization formula, Theorem 3.24, to give explicit examples of functions in $\mathcal {S}(r\cdot \mathbb {G})$.

### Example 5.1

Let $r\in (0,1)$, let $\mathcal {M}=\mathbb {C}^{2}$ and let *U* be the unitary operator on $\mathbb {C}^{2}$ given by

$$U=\begin{bmatrix} \omega _{1} &  0 \\ 0 &  \omega _{2} \\ \end{bmatrix}$$

for some $\omega _{1},\omega _{2}\in \mathbb {T}$. Let $a\in \mathbb {C}$, let $\gamma , \beta$ be vectors in $\mathbb {C}^{2}$, and let $D=u\otimes v$ be an operator on $\mathbb {C}^{2}$, where *u*, *v* are vectors in $\mathbb {C}^{2}$ with $\Vert u\Vert _{\mathbb {C}^{2}}=\Vert v\Vert _{\mathbb {C}^{2}}$. Let the operator

5.2 $$\begin{aligned} L=\begin{bmatrix} a &  1\otimes \beta \\ \gamma \otimes 1 &  D \end{bmatrix} \end{aligned}$$

be unitary on $\mathbb {C}\oplus \mathbb {C}^{2}$. Note that since *L* is unitary, the following conditions on $a, \gamma , \beta , u, v$ are satisfied

1. $$a=0$$;
2. $$\Vert \gamma \Vert =\Vert \beta \Vert =1$$;
3. $$\Vert u\Vert =\Vert v\Vert =1$$;
4. $\{\gamma ,u\}$ and $\{\beta ,v\}$ are orthonormal bases of $\mathbb {C}^{2}$.

Then, by Theorem 3.24,

5.3 $$\begin{aligned} f(s)=a+\langle s_{U,\mathcal {R}}(1_{\mathbb {C}^{2}}-Ds_{U,\mathcal {R}})^{-1}\gamma , \beta \rangle _{\mathbb {C}^{2}}, \ \text {for all} \ s\in r\cdot \mathbb {G}, \end{aligned}$$

belongs to $\mathcal {S}(r\cdot \mathbb {G})$. Here $s_{U,\mathcal {R}}$ is defined by equation (3.27). Let us show that in this case, the function *f* can be expressed by the following formula

5.4 $$\begin{aligned} f(s)=\dfrac{\Bigg \langle \begin{bmatrix} \varphi _{\omega _{1}}(s)(1-u_{2}\overline{v_{2}}r^{-1}\varphi _{\omega _{2}r^{-1}}(s)) &  r^{-1}u_{1}\overline{v_{2}}\varphi _{\omega _{1}}(s) \varphi _{\omega _{2}r^{-1}}(s)\\ r^{-1}u_{2}\overline{v_{1}}\varphi _{\omega _{1}}(s)\varphi _{\omega _{2}r^{-1}}(s) &  r^{-1}\varphi _{\omega _{2}r^{-1}}(s) (1-u_{1}\overline{v_{1}} \varphi _{\omega _{1}}(s))\\ \end{bmatrix}\gamma ,\beta \Bigg \rangle _{\mathbb {C}^{2}}}{1-u_{1}\overline{v_{1}}\varphi _{\omega _{1}}(s)-u_{2}\overline{v_{2}} r^{-1}\varphi _{\omega _{2}r^{-1}}(s)} \end{aligned}$$

for all $s\in r\cdot \mathbb {G}$. Here, for $s=(s_1,s_2)$,

5.5 $$\begin{aligned} \varphi _{z}(s) = \dfrac{s_{2}z-\frac{1}{2}s_{1}}{1-\frac{1}{2}s_{1}z}~~\text {for}~z\in \mathbb {C}~\text {such that}~1-\frac{1}{2}s_{1}z\ne 0. \end{aligned}$$

### Proof

To use Theorem 3.24, we have to be sure that all the parameters given above ensure that the matrix

5.6 $$\begin{aligned} L=\begin{bmatrix} a &  1\otimes \beta \\ \gamma \otimes 1 &  D \end{bmatrix} \end{aligned}$$

is unitary on $\mathbb {C}\oplus \mathbb {C}^{2}$, that is,

5.7 $$\begin{aligned} LL^{*}=L^{*}L=I_{\mathbb {C}\oplus \mathbb {C}^{2}}. \end{aligned}$$

We have

5.8 $$\begin{aligned} LL^{*} = \begin{bmatrix} |a|^{2}+\Vert \beta \Vert ^{2}_{\mathbb {C}^{2}} &  a\otimes \gamma +(1\otimes \beta )D^{*}\\ \overline{a}(\gamma \otimes 1)+D(\beta \otimes 1) &  \gamma \otimes \gamma +DD^{*} \end{bmatrix} \end{aligned}$$

and

5.9 $$\begin{aligned} L^{*}L = \begin{bmatrix} |a|^{2}+\Vert \gamma \Vert ^{2}_{\mathbb {C}^{2}} &  \overline{a}\otimes \beta +(1\otimes \gamma )D\\ a(\beta \otimes 1)+D^{*}(\gamma \otimes 1) &  \beta \otimes \beta +D^{*}D \end{bmatrix}. \end{aligned}$$

Since *L* is unitary, using equations (5.8) and (5.9), we can obtain the following system of equations for $a, \gamma , \beta , u, v$.

5.10 $$\begin{aligned} 1= &   |a |^{2}+\Vert \beta \Vert ^{2} = |a |^{2}+\Vert \gamma \Vert ^{2} \end{aligned}$$

5.11 $$\begin{aligned} 0= &   a\otimes \gamma +\langle v,\beta \rangle _{\mathbb {C}^{2}}(1\otimes u) = \overline{a}\otimes \beta +\langle u,\gamma \rangle _{\mathbb {C}^{2}}(1\otimes v) \end{aligned}$$

5.12 $$\begin{aligned} 0= &   \overline{a}\gamma +\langle \beta ,v\rangle _{\mathbb {C}^{2}}u = a\beta + \langle \gamma ,u \rangle _{\mathbb {C}^{2}} v \end{aligned}$$

5.13 $$\begin{aligned} 1_{\mathbb {C}^{2}}= &   \gamma \otimes \gamma + \Vert v\Vert _{\mathbb {C}^{2}}^{2}(u\otimes u) = \beta \otimes \beta + \Vert u\Vert _{\mathbb {C}^{2}}^{2}(v\otimes v). \end{aligned}$$

We claim that this system of equations forces:

1. $$a=0$$;
2. $$\Vert \gamma \Vert =\Vert \beta \Vert =1$$;
3. $$\Vert u\Vert =\Vert v\Vert =1$$;
4. $\{\gamma ,u\}$ and $\{\beta ,v\}$ are orthonormal bases of $\mathbb {C}^{2}$.

We prove statement (1) by contradiction. Suppose that $a\ne 0$. From equation (5.12),

$$0=a\beta +\langle \gamma , u\rangle _{\mathbb {C}^{2}}v.$$

Thus,

$$\beta =-a^{-1}\langle \gamma ,u\rangle _{\mathbb {C}^{2}}v$$

and

$$\beta \otimes \beta = a^{-2}|\langle \gamma ,u\rangle _{\mathbb {C}^{2}}|^{2}(v\otimes v).$$

From equation (5.13) with the expression for $\beta \otimes \beta$ above, we have

$$1_{\mathbb {C}^{2}} = (a^{-2}|\langle \gamma ,u\rangle _{\mathbb {C}^{2}}|^{2}+\Vert u\Vert _{\mathbb {C}^{2}}^{2})(v\otimes v).$$

This is a contradiction, as $v\otimes v$ is a rank 1 matrix on $\mathbb {C}^{2}$ and $1_{\mathbb {C}^{2}}$ has rank 2. Thus $a=0$ necessarily.

Statement (2) follows from equation (5.10), since $a=0$, $\Vert \gamma \Vert _{\mathbb {C}^{2}}=\Vert \beta \Vert _{\mathbb {C}^{2}}=1$. Moreover, equation (5.11) becomes

$$0 = \langle v,\beta \rangle _{\mathbb {C}^{2}}(1\otimes u) = \langle u,\gamma \rangle _{\mathbb {C}^{2}}(1\otimes v). $$

By the equation above, for all $x\in \mathbb {C}^{2}$,

5.14 $$\begin{aligned} 0= &   \langle v,\beta \rangle _{\mathbb {C}^{2}}\langle x,u\rangle _{\mathbb {C}^{2}} \end{aligned}$$

5.15 $$\begin{aligned} 0= &   \langle u,\gamma \rangle _{\mathbb {C}^{2}}\langle x,v\rangle _{\mathbb {C}^{2}}. \end{aligned}$$

Equation (5.15) implies *u* is orthogonal to $\gamma$ and equation (5.14) implies *v* is orthogonal to $\beta$. Together, $\{\gamma ,u\}$ and $\{\beta ,v\}$ are respectively orthogonal in $\mathbb {C}^{2}$. In fact, $\{\gamma ,u\}$ and $\{\beta ,v\}$ are orthonormal bases of $\mathbb {C}^{2}$; indeed, by equation (5.13), for all $x\in \mathbb {C}^{2}$,

5.16 $$\begin{aligned} x= &   \langle x,\beta \rangle _{\mathbb {C}^{2}}\beta + \Vert u\Vert _{\mathbb {C}^{2}}^{2}\langle x,v \rangle _{\mathbb {C}^{2}} v\end{aligned}$$

5.17 $$\begin{aligned} x= &   \langle x,\gamma \rangle _{\mathbb {C}^{2}}\gamma + \Vert v\Vert _{\mathbb {C}^{2}}^{2}\langle x,u\rangle _{\mathbb {C}^{2}}u. \end{aligned}$$

Let $x=v$ in equation (5.16), we have

$$v=\Vert u\Vert _{\mathbb {C}^{2}}^{2}\Vert v\Vert ^{2}_{\mathbb {C}^{2}}v.$$

By the assumption $\Vert u\Vert _{\mathbb {C}^{2}}=\Vert v\Vert _{\mathbb {C}^{2}}$ and by the equation above,

$$1=\Vert u\Vert _{\mathbb {C}^{2}}\Vert v\Vert _{\mathbb {C}^{2}} = \Vert u\Vert _{\mathbb {C}^{2}}^{2}=\Vert v\Vert _{\mathbb {C}^{2}}^{2}.$$

Therefore $$\Vert u\Vert _{\mathbb {C}^{2}}=\Vert v\Vert _{\mathbb {C}^{2}}=1$$.

We can now utilise the realization formula (5.3)

5.18 $$\begin{aligned} f(s)=a+\langle s_{U,\mathcal {R}}(1_{\mathbb {C}^{2}}-Ds_{U,\mathcal {R}})^{-1}\gamma , \beta \rangle _{\mathbb {C}^{2}}, \ \text {for all} \ s\in r\cdot \mathbb {G}. \end{aligned}$$

Under our assumptions, we have shown that *a* has to be equal to 0. By assumption,

$$D =u\otimes v = \begin{bmatrix} u_{1}\overline{v_{1}} &  u_{1}\overline{v_{2}} \\ u_{2}\overline{v_{1}} &  u_{2}\overline{v_{2}} \\ \end{bmatrix}.$$

For $$U=\begin{bmatrix} \omega _{1} &  0 \\ 0 &  \omega _{2} \\ \end{bmatrix}$$ and for $s= (s_1, s_2) \in r\cdot \mathbb {G}$,

5.19 $$\begin{aligned} s_{U,\mathcal {R}}= &   \bigg (2s_{2}\mathcal {R}^{-1}U-s_{1}\bigg )\bigg (2\mathcal {R}-s_{1}U\bigg )^{-1}\end{aligned}$$

5.20 $$\begin{aligned}= &   \begin{bmatrix} \dfrac{s_{2} \omega _1-\frac{1}{2}s_{1}}{1-\frac{1}{2}s_{1} \omega _1} &  0 \\ 0 &  r^{-1} \dfrac{s_{2}\omega _{2}r^{-1} -\frac{1}{2}s_{1}}{1-\frac{1}{2}s_{1} \omega _{2}r^{-1} } \\ \end{bmatrix}. \end{aligned}$$

Let us use the notation

$$ \varphi _{z}(s) = \dfrac{s_{2}z-\frac{1}{2}s_{1}}{1-\frac{1}{2}s_{1}z} \ \text {for} \ z\in \mathbb {C} \ \text {such that}~1-\frac{1}{2}s_{1}z\ne 0. $$

Thus, for $$s= (s_1, s_2) \in r\cdot \mathbb {G}$$,

$$ s_{U,\mathcal {R}} = \begin{bmatrix} \varphi _{\omega _1}(s) &  0 \\ 0 &  r^{-1} \varphi _{r^{-1}\omega _2}(s) \\ \end{bmatrix}. $$

Therefore

$$1_{\mathbb {C}^{2}}-( u\otimes v )s_{U,\mathcal {R}} = \begin{bmatrix} 1-u_{1}\overline{v_{1}}\varphi _{\omega _{1}}(s) &  -u_{1}\overline{v_{2}}r^{-1}\varphi _{\omega _{2}r^{-1}}(s)\\ -u_{2}\overline{v_{1}}\varphi _{\omega _{1}}(s) &  1-u_{2}\overline{v_{2}}r^{-1}\varphi _{\omega _{2}r^{-1}}(s) \end{bmatrix}.$$

Note that

$$\textrm{det}(1_{\mathbb {C}^{2}}-( u\otimes v )s_{U,\mathcal {R}}) =1-u_{1}\overline{v_{1}}\varphi _{\omega _{1}}(s)-u_{2}\overline{v_{2}}r^{-1}\varphi _{\omega _{2}r^{-1}}(s).$$

Hence, so long as $\textrm{det}(1_{\mathbb {C}^{2}}-( u\otimes v )s_{U,\mathcal {R}})\ne 0$, $1_{\mathbb {C}^{2}}-( u\otimes v )s_{U,\mathcal {R}}$ is invertible and is given by

$$\begin{aligned} (1_{\mathbb {C}^{2}}&-( u\otimes v )s_{U,\mathcal {R}})^{-1} \\&= \big [\textrm{det}(1_{\mathbb {C}^{2}}-( u\otimes v )s_{U,\mathcal {R}})\big ]^{-1}\begin{bmatrix} 1-u_{2}\overline{v_{2}}r^{-1}\varphi _{\omega _{2}r^{-1}}(s) &  u_{1}\overline{v_{2}}r^{-1}\varphi _{\omega _{2}r^{-1}}(s)\\ u_{2}\overline{v_{1}}\varphi _{\omega _{1}}(s) &  1-u_{1}\overline{v_{1}}\varphi _{\omega _{1}}(s) \end{bmatrix} \\&=\dfrac{\begin{bmatrix} 1-u_{2}\overline{v_{2}}r^{-1}\varphi _{\omega _{2}r^{-1}}(s) &  u_{1}\overline{v_{2}}r^{-1}\varphi _{\omega _{2}r^{-1}}(s) \\ u_{2}\overline{v_{1}}\varphi _{\omega _{1}}(s) &  1-u_{1}\overline{v_{1}}\varphi _{\omega _{1}}(s) \end{bmatrix}}{1-u_{1}\overline{v_{1}}\varphi _{\omega _{1}}(s) -u_{2}\overline{v_{2}}r^{-1}\varphi _{\omega _{2}r^{-1}}(s) }. \end{aligned}$$

Therefore, the function *f* given by equation (5.18) is defined by

$$\begin{aligned} f(s)=\dfrac{\Bigg \langle \begin{bmatrix} \varphi _{\omega _{1}}(s) (1-u_{2}\overline{v_{2}}r^{-1}\varphi _{\omega _{2}r^{-1}}(s) ) &  r^{-1}u_{1}\overline{v_{2}}\varphi _{\omega _{1}}(s) \varphi _{\omega _{2}r^{-1}}(s) \\ r^{-1}u_{2}\overline{v_{1}}\varphi _{\omega _{1}}(s) \varphi _{\omega _{2}r^{-1}}(s) &  r^{-1}\varphi _{\omega _{2}r^{-1}}(s) (1-u_{1}\overline{v_{1}}\varphi _{\omega _{1}}(s) )\\ \end{bmatrix}\gamma ,\beta \Bigg \rangle _{\mathbb {C}^{2}}}{1-u_{1}\overline{v_{1}}\varphi _{\omega _{1}}(s) -u_{2}\overline{v_{2}}r^{-1}\varphi _{\omega _{2}r^{-1}}(s) } \end{aligned}$$

for all $s\in r\cdot \mathbb {G}$. By Theorem 3.24, this function *f* belongs to $\mathcal {S}(r\cdot \mathbb {G})$.$\square$

### Example 5.21

For any $r\in (0,1)$ and $\omega \in \mathbb {T}$, the function $\Upsilon _{\omega ,r}$ defined by

5.22 $$\begin{aligned} \Upsilon _{\omega ,r}(s)=\dfrac{s_{2}\omega r^{-1}-\dfrac{1}{2}s_{1}}{1-\dfrac{1}{2}s_{1}\omega r^{-1}}r^{-1}, \ \text {for all} \ s=(s_{1},s_{2})\in r\cdot \mathbb {G}, \end{aligned}$$

belongs to $$\mathcal {S}(r\cdot \mathbb {G})$$.

### Proof

In Example 5.1 take $\omega _{1}=\omega _{2}=\omega$ to be complex numbers on the unit circle and the vectors $\beta =\gamma =e_{2}$ and $u=v=e_{1}$, where

$$e_{1}=\begin{bmatrix} 1 \\ 0 \end{bmatrix},~e_{2}=\begin{bmatrix} 0 \\ 1 \end{bmatrix},$$

the standard orthonormal bases in $\mathbb {C}^{2}$. Then $\Upsilon _{\omega ,r}\in \mathcal {S}(r\cdot \mathbb {G})$ and has the form given in equation (5.22). $\square$

The next example gives us $\Phi _{\omega }$ with $\omega \in \mathbb {T}$, which is the familiar “magic function” for $\mathbb {G}$, see Agler and Young [2]. The functions $\Phi _{\omega }$, $\omega \in \mathbb {T}$, where $\Phi _{\omega }(s,p)= \dfrac{2\omega p-s}{2-\omega s}$ for $(s,p)\in \mathbb {G}$, were called “magic functions” by Agler in recognition of their power as a tool to prove facts about $\mathbb {G}$. The main application of magic functions in [2, 3], was to identify all automorphisms of $\mathbb {G}$, and they are also central to the solution of the Carathéodory extremal problem for $\mathbb {G}$.

Note that $\Upsilon _{\omega ,r}$ from Example 5.21 reduces to the equation

5.23 $$\begin{aligned} \Upsilon _{\omega ,r}(s)=\Phi _{\omega r^{-1}}(s)r^{-1} \ \text {for all} \ s=(s_{1},s_{2})\in r\cdot \mathbb {G}. \end{aligned}$$

### Example 5.24

For any $\omega \in \mathbb {T}$, the function defined by

$$\Phi _{\omega }(s) = \dfrac{s_{2}\omega -\frac{1}{2}s_{1}}{1-\frac{1}{2}s_{1}\omega }$$

for all $s=(s_{1},s_{2})\in \mathbb {G}$, belongs to $\mathcal {S}(\mathbb {G})$, and so to $\mathcal {S}(r\cdot \mathbb {G})$.

### Proof

In Example 5.1, take $\omega _{1}=\omega _{2}=\omega$ to be a complex number on the unit circle, $\gamma =\beta =e_{1}$ and $u=v=e_{2}$, the standard basis of $\mathbb {C}^{2}$. Then the description of the function *f* from equation (5.4) gives us

$$\begin{aligned} f(s)&=\dfrac{\Bigg \langle \begin{bmatrix} \varphi _{\omega }(s) (1-r^{-1}\varphi _{\omega r^{-1}}(s) ) &  0\\ 0 &  0\\ \end{bmatrix}e_{1},e_{1}\Bigg \rangle _{\mathbb {C}^{2}}}{(1-r^{-1}\varphi _{\omega r^{-1}}(s) )}\\&=\dfrac{\varphi _{\omega }(s)(1-r^{-1}\varphi _{\omega r^{-1}}(s) )}{(1-r^{-1}\varphi _{\omega r^{-1}}(s) )} \\&= \varphi _{\omega }(s) = \dfrac{s_{2}\omega -\frac{1}{2}s_{1}}{1-\frac{1}{2}s_{1}\omega }\\&= \Phi _{\omega }(s), \end{aligned}$$

for all $s=(s_{1},s_{2})\in r\cdot \mathbb {G}$. It is well known that this function is well defined on $\mathbb {G}$ and belongs to $\mathcal {S}(\mathbb {G})$.$\square$

### Example 5.25

For any $\omega _{1},\omega _{2}\in \mathbb {T}$ and $r\in (0,1)$, the function

$$f(s)=\dfrac{r-\sqrt{2}\varphi _{\omega _{2}r^{-1}}(s) }{r\sqrt{2}-\varphi _{\omega _{2}r^{-1}}(s) }\varphi _{\omega _{1}}(s) \ \text {for all} \ s=(s_{1},s_{2})\in r\cdot \mathbb {G}, $$

belongs to $$\mathcal {S}(r\cdot \mathbb {G})$$.

### Proof

Suppose that

$$\gamma =\dfrac{1}{\sqrt{2}}\begin{bmatrix} 1 \\ 1 \end{bmatrix}, ~u=\dfrac{1}{\sqrt{2}}\begin{bmatrix} -1 \\ 1 \end{bmatrix}$$

and $\beta =e_{1}$, $v=e_{2}$ for the standard basis $e_{1},e_{2}$ of $\mathbb {C}^{2}$. By Example 5.1, the function

$$f(s)=\dfrac{r-\sqrt{2}\varphi _{\omega _{2}r^{-1}}(s)}{r\sqrt{2}-\varphi _{\omega _{2}r^{-1}}(s)}\varphi _{\omega _{1}}(s),$$

where $s\in r\cdot \mathbb {G}$ and $\varphi _{z}$ is given by formula (5.5), belongs to $\mathcal {S}(r\cdot \mathbb {G})$.$\square$

## Data Availability

No data were collected, generated or consulted in connection with this research.
